# *Mycobacterium tuberculosis* Universal Stress Protein Rv2623 Regulates Bacillary Growth by ATP-Binding: Requirement for Establishing Chronic Persistent Infection

**DOI:** 10.1371/journal.ppat.1000460

**Published:** 2009-05-29

**Authors:** Joshua E. Drumm, Kaixia Mi, Patrick Bilder, Meihao Sun, Jihyeon Lim, Helle Bielefeldt-Ohmann, Randall Basaraba, Melvin So, Guofeng Zhu, JoAnn M. Tufariello, Angelo A. Izzo, Ian M. Orme, Steve C. Almo, Thomas S. Leyh, John Chan

**Affiliations:** 1 Department of Medicine, Albert Einstein College of Medicine, Bronx, New York, United States of America; 2 Department of Microbiology & Immunology, Albert Einstein College of Medicine, Bronx, New York, United States of America; 3 Department of Biochemistry, Albert Einstein College of Medicine, Bronx, New York, United States of America; 4 Department of Physiology & Biophysics, Albert Einstein College of Medicine, Bronx, New York, United States of America; 5 Mycobacteria Research Laboratories, Department of Microbiology, Immunology and Pathology, Colorado State University, Fort Collins, Colorado, United States of America; University of Washington, United States of America

## Abstract

Tuberculous latency and reactivation play a significant role in the pathogenesis of tuberculosis, yet the mechanisms that regulate these processes remain unclear. The *Mycobacterium tuberculosis* universal stress protein (USP) homolog, *rv2623*, is among the most highly induced genes when the tubercle bacillus is subjected to hypoxia and nitrosative stress, conditions thought to promote latency. Induction of *rv2623* also occurs when *M. tuberculosis* encounters conditions associated with growth arrest, such as the intracellular milieu of macrophages and in the lungs of mice with chronic tuberculosis. Therefore, we tested the hypothesis that Rv2623 regulates tuberculosis latency. We observed that an Rv2623-deficient mutant fails to establish chronic tuberculous infection in guinea pigs and mice, exhibiting a hypervirulence phenotype associated with increased bacterial burden and mortality. Consistent with this *in vivo* growth-regulatory role, constitutive overexpression of *rv2623* attenuates mycobacterial growth *in vitro*. Biochemical analysis of purified Rv2623 suggested that this mycobacterial USP binds ATP, and the 2.9-Å-resolution crystal structure revealed that Rv2623 engages ATP in a novel nucleotide-binding pocket. Structure-guided mutagenesis yielded Rv2623 mutants with reduced ATP-binding capacity. Analysis of mycobacteria overexpressing these mutants revealed that the *in vitro* growth-inhibitory property of Rv2623 correlates with its ability to bind ATP. Together, the results indicate that i) *M. tuberculosis* Rv2623 regulates mycobacterial growth *in vitro* and *in vivo*, and ii) Rv2623 is required for the entry of the tubercle bacillus into the chronic phase of infection in the host; in addition, iii) Rv2623 binds ATP; and iv) the growth-regulatory attribute of this USP is dependent on its ATP-binding activity. We propose that Rv2623 may function as an ATP-dependent signaling intermediate in a pathway that promotes persistent infection.

## Author Summary

*Mycobacterium tuberculosis* poses serious threats to public health worldwide. The ability of this pathogen to establish in the host a clinically silent, persistent latent infection that can subsequently reactivate to cause diseases constitutes a major challenge in controlling tuberculosis. Our study showed that an *M. tuberculosis* mutant that is deficient in a universal stress protein (USP) designated Rv2623 fails to establish a chronic persistent infection in animal hosts. The mutant strain exhibits a hypervirulent phenotype as assessed by increased bacillary growth, pathology, and mortality in infected animals relative to the parental strain. Consistent with this *in vivo* growth-regulating attribute, we demonstrated that Rv2623, when expressed in mycobacteria at levels higher than that of the wild-type strain, retards bacterial growth *in vitro*. Using biochemical and biophysical analyses, including the Rv2623 crystal structure, we showed that this USP binds to ATP within a novel ATP-binding pocket. Through targeted mutagenesis studies, we further determined that the ability of Rv2623 to regulate bacillary growth is dependent on its ATP-binding capacity. Our data strongly suggest Rv2623 as a critical component that regulates the entry of *M. tuberculosis* into a chronic persistent growth phase, and therefore provide valuable insight into tuberculous dormancy and uncover new opportunities for the development of novel anti-tuberculous therapies.

## Introduction

*Mycobacterium tuberculosis*, one of the most successful human pathogens, infects one-third of the world's population, causing nearly two million deaths per year [1]. Epidemiological data estimate that, in the immunocompetent host, only ∼10% of *M. tuberculosis* infection progress to active pulmonary disease. The remaining 90% of the infected individuals are asymptomatic, and are generally believed to harbor latent bacilli that can reactivate to cause tuberculous diseases, sometimes decades after the initial infection. Recrudescence of latent bacilli contributes significantly to the incidence of adult tuberculosis [2], yet the physiological state of latent bacilli and the signals that promote dormancy in the host remain incompletely defined. Understanding the dynamic interaction between host and pathogen during the establishment of persistent *M. tuberculosis* infection will guide the design of novel treatment for the latently infected population.

An intracellular pathogen, *M. tuberculosis* must possess a finely tuned signaling network to sense and transduce complex environmental signals, ensuring survival of the bacilli within host cells. Nitric oxide (NO) produced by infected macrophages and relative hypoxia are signals likely to be encountered within tuberculous lesions that are believed, based on *in vitro* studies, to promote latency by prompting the *M. tuberculosis* dormancy response. Exposure to these stimuli results in the induction of ∼50 *M. tuberculosis* genes, designated the dormancy regulon, via the two-component regulatory system DosR-DosS (see Table S1 for accession numbers) [3],[4],[5]. Among this set of genes is *rv2623*, one of eight *M. tuberculosis* genes annotated as containing the universal stress protein (USP) domain [6],[7]. Members of this ancient and conserved family of proteins are found in all forms of life and can be induced by a variety of environmental stresses [8],[9]. However, the roles of USP proteins in microbial pathogenesis are incompletely understood.

Interestingly, *rv2623* is one of the most strongly induced transcripts of the dormancy regulon [3],[4],[5]. Increased expression of *rv2623* was also observed following phagocytosis by macrophages [10] and in the lungs of chronically infected mice [11], supporting a functional role during persistent *M. tuberculosis* infection. The present study reveals that: i) deletion of *rv2623* confers hypervirulence on the tubercle bacillus in animal models, suggesting that expression of Rv2623 may be conducive to the establishment of persistence *in vivo*; ii) overexpression of Rv2623 results in growth retardation of recipient strains *in vitro*, further supporting a growth-regulatory role; iii) Rv2623 binds ATP; and finally, through mutagenesis study guided by crystallographic analysis of Rv2623 (the first such study for a tandem-domain USP), we show that iv) the growth-regulating attribute of this *M. tuberculosis* USP is linked to its ATP-binding capacity.

## Results

### *Mycobacterium tuberculosis* Rv2623 regulates growth *in vitro*

**Figure 1:**
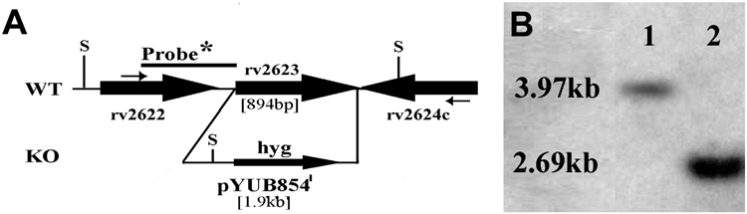
Generation of Δ*rv2623 M. tuberculosis* strain. (A) Genomic organization of the *rv2623* gene locus. Genes appear as large arrows in their native orientation. Small arrows represent forward and reverse primers used for long range PCR. Sizes of the *rv2623* deletion and *hyg*-insertion are indicated. The location of the radiolabeled probe (black bar) as well as relevant Sph1 sites (S) is indicated as they pertain to the Southern blot. (B) An autoradiograph of the Southern blot shows radiolabeled Sph1 fragments from the wild type (lane 1) and Δ*rv2623* strain (lane 2). The sizes indicated represent those expected for wild type and the deletion mutant.

An *rv2623*-deletion mutant of the virulent *M. tuberculosis* Erdman strain was generated by specialized transduction [12]. The *rv2623*-specific allelic exchange construct was delivered via recombinant mycobacteriophage phAE159 and transformants were analyzed by Southern blot, confirming replacement of *rv2623* with the *hyg* gene, which confers hygromycin resistance (Figure 1A). Aliquots of a single knockout clone, designated as Δ*rv2623*, were stored at −70°C. Deletion of *rv2623* is not likely to affect transcription of neighboring genes, given the sequence-confirmed precise excision of the *rv2623* coding region and the gene organization at the *rv2623* locus (the downstream *rv2624c* is transcribed in the direction opposite to that of *rv2623*) (Figure 1B).

**Figure 2:**
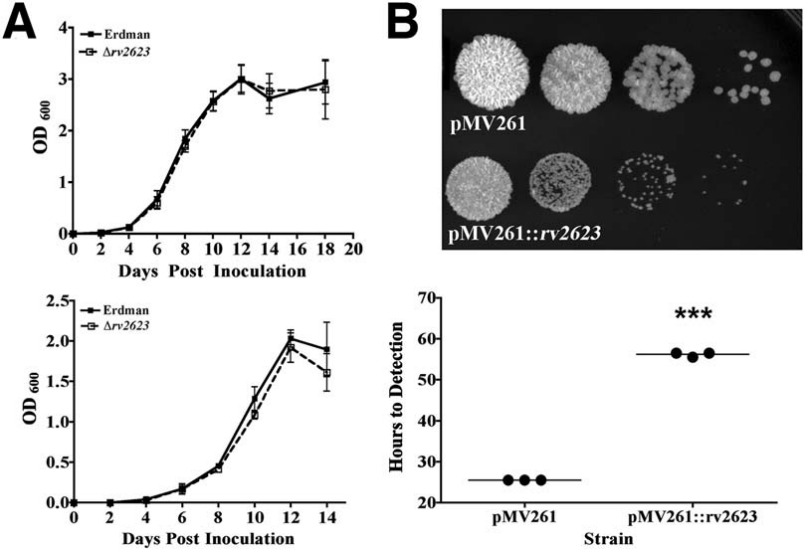
*In vitro* characterization of Δ*rv2623*. (A) Growth curves of cultures inoculated (10^6^ CFU/ml) into 7H9+10% OADC+0.05% Tween 80 (top) and in minimal Sauton's media (bottom); Erdman (closed boxes, solid line) and Δ*rv2623* (open boxes, dashed line) cultures. Error bars represent the standard error of the means; each curve is a combination of at least three independent experiments. (B) Overexpression of Rv2623 in *M. smegmatis*. The top panel represents serial dilutions (1∶10) of the empty vector pMV261-containing negative control strain and Rv2623-overexpressing strain harboring pMV261::*rv2623*. Diluted stationary phase *M. smegmatis* culture was spotted (5 µl) onto solid 7H10 media supplemented with 10% OADC and kanamycin (40 µg/mL). Photographs were taken after three days incubation at 37°C. Growth of corresponding strains in liquid medium was assessed based on the time to detection determined using a BD BACTEC 9000MB system (bottom). The various strains were inoculated at 10^4^ CFU/ml in triplicates. Data shown are representative of several independent experiments. ***p<0.001.

Deletion of specific USPs in *E. coli* results in growth defects *in vitro* [8],[13],[14]. For example, an *E. coli* strain deficient for UspA exhibits reduced survival in stationary phase culture [14]. However, the *in vitro* growth kinetics of Δ*rv2623 M. tuberculosis* in OADC-supplemented Middlebrook 7H9 or minimal Sauton's medium is comparable to that of wildtype Erdman up to 14 days post-inoculation (Figure 2A). We reasoned that a potential growth-regulating attribute of Rv2623 might be masked by functional redundancy among the *M. tuberculosis* USP homologs. Indeed, partial functional overlap has been demonstrated among the *E. coli* USPs [9],[15]. We therefore examined the effect of overexpression of this USP in the rapidly growing *M. smegmatis* strain mc^2^155 [16]. As seen in Figure 2B, constitutive overexpression of *M. tuberculosis rv2623* using the multi-copy plasmid pMV261 resulted in growth deficiency of the recipient strain both on solid medium (Middlebrook 7H10 agar) and in the liquid medium-based BD BACTEC 9000MB system. These results strongly suggest that *M. tuberculosis* Rv2623 regulates mycobacterial growth *in vitro*.

### Rv2623 regulates mycobacterial growth *in vivo*

**Figure 3:**
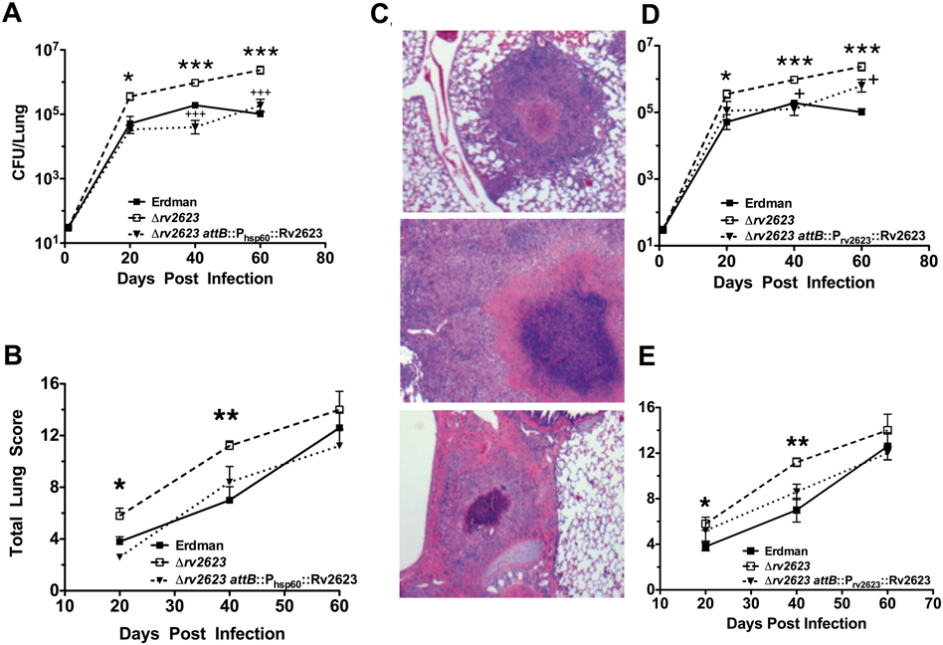
*In Vivo* growth of and pathology caused by Δ*rv2623* in guinea pigs. Outbred Hartley guinea pigs given an aerosol challenge of ∼30 CFU were assessed for pulmonic bacterial burden (A,D) and the severity of lung pathology (B,E). Closed box, open box, and triangle represent guinea pigs infected with Erdman, in (A,B,D,E), Δ*rv2623*, in (A,B,D,E), and Δ*rv2623 attB*::P_hsp60_ Rv2623, in (A,B), or Δ*rv2623 attB*::P_rv2623_ Rv2623, in (D,E). Comparing the wildtype Erdman and the Δ*rv2623* strains: *p<0.05; **p<0.01; ***p<0.001. Comparing the Δ*rv2623* and the Δ*rv2623*::complemented strains (Δ*rv2623 attB*::P_hsp60_ Rv2623 or Δ*rv2623 attB*::P_rv2623_ Rv2623): ^+++^p<0.001; ^+^p<0.05. (C) Hematoxylin & Eosin-stained lung sections (40 days post infection) from guinea pigs infected with Erdman (top), Δ*rv2623* (middle), and Δ*rv2623 attB*::P_hsp60_ Rv2623 (bottom) *M. tuberculosis*. Error bars represent the standard error of the mean.

Although USP family proteins are expressed by many bacterial pathogens [7],[8], to date, there has only been one *in vivo* study, which showed that a Salmonella USP promotes virulence in mice [17]. The observation that Rv2623 modulates mycobacterial growth *in vitro* prompted us to examine the effect of this USP on the *in vivo* kinetics of *M. tuberculosis* infection. Low dose aerosol infection of outbred Hartley guinea pigs with ∼30 CFU revealed a clear growth advantage of the Δ*rv2623* mutant strain relative to wildtype. As early as 20 days post-infection, the number of *M. tuberculosis* bacilli present in the lungs of Δ*rv2623*-infected guinea pigs was ∼10-fold higher (p<0.05) than those infected with wildtype Erdman, and continued to rise, attaining a 15-fold (p<0.001) difference by 60 days post-infection (Figure 3A). Guinea pigs are able to control the growth of Erdman bacilli following the onset of adaptive immunity at ∼3 weeks post-infection, as evident by the relatively stable pulmonary bacterial burden beyond the 3 week time point, yet levels of Δ*rv2623* bacilli continued to increase at a reduced but steady rate resulting in a rapidly progressing infection. Moreover, Δ*rv2623*-infected guinea pigs were moribund at 60 days post-infection, while those challenged with wildtype Erdman remained relatively healthy, providing further evidence that the mutant strain is hypervirulent in this model. Finally, complementation with a single integrated copy of *rv2623* expressed from a constitutive mycobacterial promoter (Δ*rv2623 attB*::P_hsp60_Rv2623) abrogated the growth advantage of the deletion mutant (Figure 3A). Also consistent with the fulminate disease progression displayed by Δ*rv2623*-infected guinea pigs are the more severe pathological changes observed as early as 20 days post-infection in the lungs of these animals, as assessed by histopathological studies, including the semi-quantitative Total Lung Score analysis (Figure 3B and Protocol S1). Overall, the progression of pulmonic lesions was accelerated in Δ*rv2623*-infected animals compared to those infected with wildtype Erdman, accompanied by more extensive necrosis and widespread fibrosis. This increase in lung pathology was also largely reversed in animals infected with the complemented Δ*rv2623 attB*::P_hsp60_Rv2623 strain (Figure 3B and C). Results of the complementation experiments were further validated using a complemented strain Δ*rv2623 attB*::P_rv2623_Rv2623, whose expression of the wildtype universal stress protein is driven by the native *rv2623* promoter [18] (Figure 3D and E).

**Figure 4:**
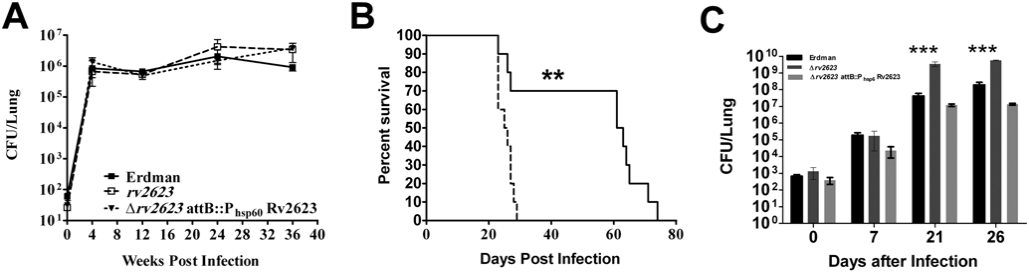
*In Vivo* growth of Δ*rv2623* in mice. (A) C57BL/6 mice infected with various strains of *M. tuberculosis* via the aerosol route with a low dose (∼100 CFU) were assessed for lung bacteria burden. Wild type Erdman (closed box, solid line); Δ*rv2623* (open box, dashed line) and the complemented strain Δ*rv2623* P_hsp60_::Rv2623 (triangle, dashed line). (B) Survival curve of C3H/HeJ mice infected via aerosol with 750–1000 CFU. Erdman and Δ*rv2623*-infected mice are represented by solid and dashed lines, respectively. (C) Kinetics of infection, established via aerolization (inoculum: ∼1,000 CFU) of wildtype Erdman (dark), Δ*rv2623* (dark grey), and the complemented strain Δ*rv2623* P_hsp60_::Rv2623 (light grey), as assessed by lung bacterial burden. **p<0.01; ***p<0.001.

In contrast to the result of the guinea pig study, we observed no difference in the kinetics of infection between C57BL/6 mice infected with wildtype *M. tuberculosis*, Δ*rv2623*, or the *attB*::P_hsp60_ Rv2623 complemented strain in a low dose aerogenic model [19], as assessed by lung bacterial burden (Figure 4A). However, the mouse is a relatively resistant host to *M. tuberculosis*, particularly in strains such as C57BL/6 [20],[21]. In fact, evidence exists that *M. tuberculosis* triggers an immune response in mice that is in excess of that required for controlling the infection [22],[23]. Thus, the hypervirulence phenotype of Δ*rv2623* observed in the susceptible guinea pig model could have been masked in the C57BL/6 mice. Consequently, we examined the virulence of Δ*rv2623* in the relatively susceptible C3H/HeJ mouse strain [24]. Indeed, the Δ*rv2623* mutant was markedly more virulent relative to wildtype Erdman *M. tuberculosis* following aerogenic infection, as assessed by the mean survival time of C3H/HeJ mice infected with these strains (62 and 25.5 days post infection for Erdman- and Δ*rv2623*-infected mice, respectively, p = 0.0014; Figure 4B). In agreement with the survival data, quantification of tissue bacterial burden revealed a growth advantage for the Rv2623-deficient mutant relative to wildtype *M. tuberculosis* Erdman (Figure 4C). Manifestation of this hypervirulence phenotype is apparent as early as 3 weeks post-infection, with the lung bacterial burden of mice infected with Δ*rv2623 M. tuberculosis* ∼100 fold higher than that in the wildtype-infected animals. As in the guinea pig studies, results of complementation experiments involving the reintroduction of a single copy of wildtype *rv2623* into Δ*rv2623 M.tuberculosis* reverses the hypervirulence (Figure 4C) exhibited in the C3H/HeJ model, thus indicating that the observed growth phenotype of the tubercle bacillus deficient for the universal stress protein is *rv2623*-specific. Finally, survival of Δ*rv2623*-infected mice was also significantly reduced in another susceptible mouse strain, C3HeB/FeJ (Figure S1). Together, the animal studies provide strong evidence that Rv2623 regulates the growth of *M. tuberculosis in vivo*: in the absence of Rv2623, the tubercle bacillus fails to establish a chronic persistent infection, exhibiting a hypervirulent phenotype.

### Effects of environmental stress on growth of *M. tuberculosis* Δ*rv2623 in vitro*

Although the functions of universal stress proteins have yet to be completely defined, there is evidence that many USPs play differential roles in protecting microbes against various environmental stresses [9]. Therefore, the hypervirulence of Δ*rv2623* in guinea pigs and susceptible mice is intriguing; if Rv2623 provides *M. tuberculosis* protection against stress, it might be expected that the Rv2623-deficient mutant would be attenuated *in vivo*. The growth kinetics and survival of the Δ*rv2623* strain was examined under various stress conditions, including those likely to be present during *M. tuberculosis* infection. These included oxidative stress (superoxide anion, O_2_^−^), DNA damage (UV irradiation, mitomycin C), heat shock (53°C), and acidic culture (pH 4.0). The use of streptonigrin, an antibiotic whose toxicity correlates with levels of free iron, was based on the observation that the intracellular environment of macrophages can induce a iron-scavenging response in mycobacteria [25], perhaps as a means of maintaining adequate levels of this important growth factor, and that an *E. coli* USP was shown to regulate iron uptake [9]. The results showed that the mutant strain was no more susceptible to growth inhibition than was wild type Erdman under all of the stress conditions tested (Figure S2). These results support the notion that it is unlikely that *M. tuberculosis* Rv2623 is essential for resistance to stresses encountered in the host, which is consistent with the observed *in vivo* hypervirulence phenotype of Δ*rv2623*.

### *M. tuberculosis* Rv2623 is a nucleotide-binding protein

We began a biochemical characterization of Rv2623 in order to gain insight into the relationship between the molecular structure/function of this USP and it's growth-regulatory properties. *M. tuberculosis* Rv2623 was expressed in *E. coli* and purified to homogeneity for biochemical studies. SDS-PAGE analysis of affinity-purified His_6_-Rv2623 revealed a single band that approximates the predicted molecular mass of ∼31.6 kDa, which was identified by immunoblotting as Rv2623 (Figure S3). Gel filtration analysis of native His_6_-Rv2623 revealed that the purified protein exists primarily as a single species with an apparent molecular mass of 61±1 kDa; suggesting that Rv2623 is a dimer under native conditions (Figure S3), an observation that was later confirmed using nano electrospray ionization (nano ESI) mass spectrometry (data not shown).

**Figure 5:**
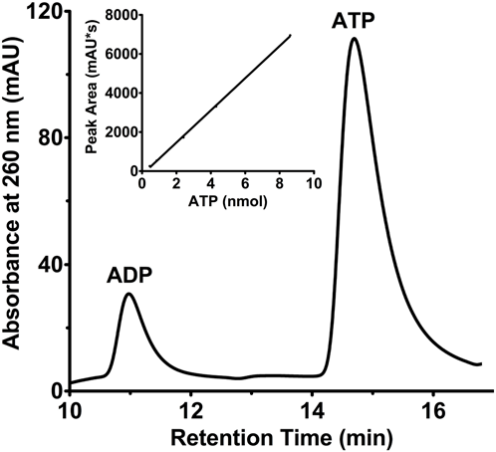
*M. tuberculosis* Rv2623 is a nucleotide-binding USP. High Performance Liquid Chromatography (HPLC) analysis of endogenously bound nucleotides from purified His_6_-Rv2623. Nucleotides species were identified based on their specific retention times on the Mono Q HR 5/5 column, represented by peaks in absorbance at 260 nm, which correspond to that of nucleotide standards (not shown). Bound nucleotides were extracted by boiling, and separated and quantified from a standard curve that relates absorbance peak area to the known amount of ATP injected onto the column (inset).

The nucleotide-binding capacity of a subset of USPs was discovered following the observation that MJ0577, a single-domain USP from *Methanococcus jannaschii*, co-purifies and co-crystallizes with ATP [26]. On the basis of structures of ATP-binding and non-ATP-binding USPs, a G-2X-G-9X-G(S/T) motif was suggested to be essential for the binding of ATP [27]. The presence of this motif in each of the two tandem USP domains of Rv2623 [7] raised the possibility that this protein possesses ATP binding activity. An HPLC-based examination of supernatants from boiled samples of His_6_-Rv2623 demonstrated that His_6_-Rv2623 co-purifies with both ATP and ADP (Figure 5). Analysis of *E. coli*-expressed Rv2623 using nano ESI mass spectrometry also demonstrated that an ATP-saturated form of dimeric Rv2623 (composed of 2 bound ATP molecules per monomer) constitutes at least half of the purified sample (data not shown). Measurement of the binding stoichiometry, which comprised HPLC-based quantification of adenine nucleotides from the boiled supernatant and spectral analysis of heat denatured Rv2623 following reconstitution in 6 M guanidine-HCl, yields 1.4±0.2 nucleotide equivalents/monomer with an overall content of 86±4% ATP (14±4% ADP). Thus, Rv2623 binds endogenous adenine nucleotides in *E. coli*, and the association is sufficiently tight that nearly 75% of the nucleotide binding sites are occupied upon purification. Indeed, nucleotide did not completely dissociate from the protein following an extensive, two-week dialysis with multiple changes against nucleotide-free buffer (approximately 0.3 nucleotide equivalents per monomer remain). It is conceivable that the presence of ADP is the consequence of an Rv2623-associated ATP activity and this putative ATPase function is currently under investigation.

### The crystal structure of *M. tuberculosis* Rv2623: Dimer assembly and ATP-binding capacity

**Figure 6:**
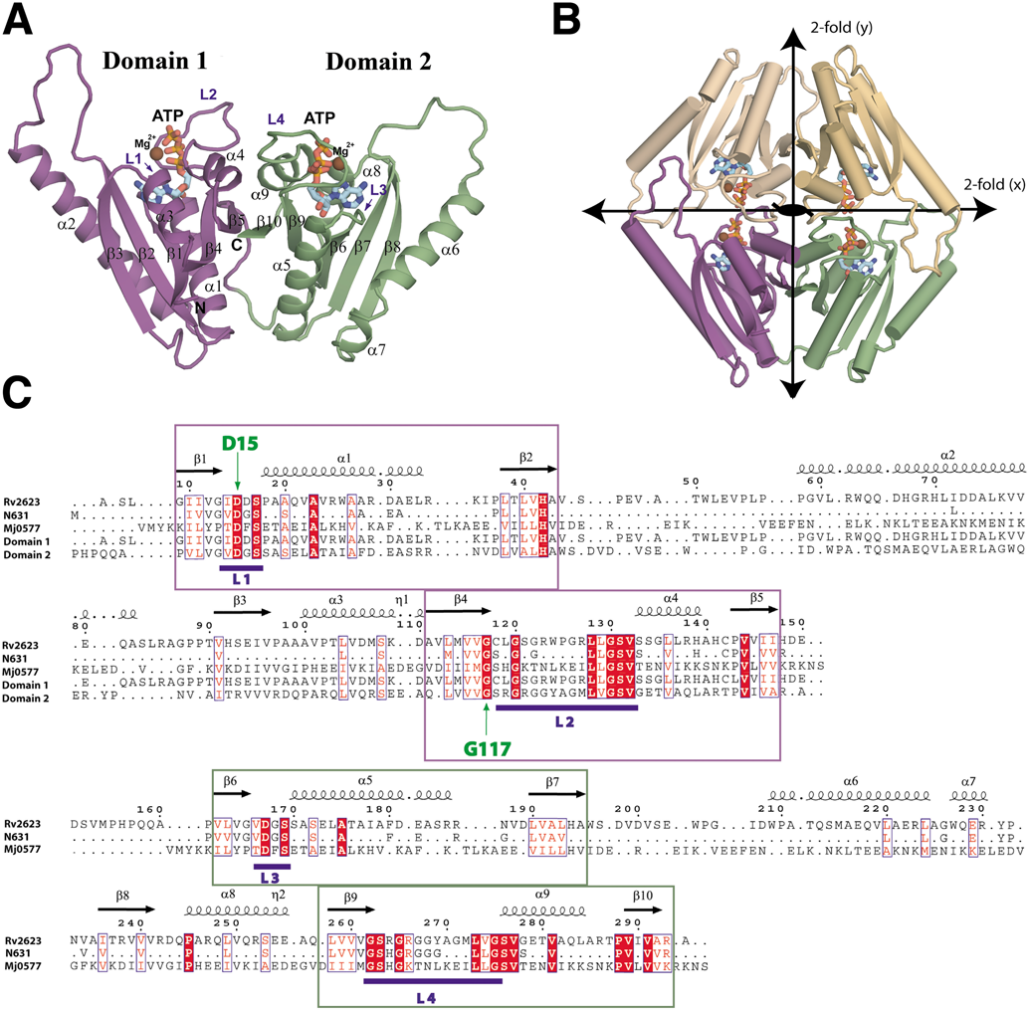
Structure and phylogeny of Rv2623 from *M. tuberculosis*. (A,B) A ribbon representation of the Rv2623 monomer (A) and dimer (B) with bound ATP (sticks) and Mg^2+^ (chocolate spheres). The three, mutually perpendicular pseudo-two-fold axes of the dimer are represented by lines with double arrows (along x, y) and a central ellipse (along z). The atoms of the bound ATP are colored cyan (carbon), red (oxygen), blue (nitrogen), and orange (phosphorus) in (A) and (B). (C) A structure-based sequence alignment of Rv2623, the N631 subfamily consensus, *Methanococcus jannaschii* protein 0577 (MJ0577), and domains 1 and 2 of Rv2623. Invariant residues in the alignment (>85% conserved in N631) are shaded in bold red and similarities are boxed in blue but left unshaded. Regions with consensus ATP binding motifs comprising L1/L2 (domain 1) and L3/L4 (domain 2) are colored dark violet and smudge, respectively. The positions of the mutated amino acids (D15, G117) are indicated in green. The structure-based sequence alignment was produced using ESPript and the structural representations were produced using PyMOL.

**Figure 7:**
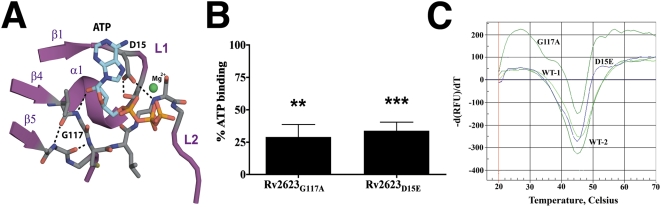
Design and stability of ATP-binding–deficient Rv2623 mutants. (A) A ribbon and stick representation of the mutation sites within the ATP binding pocket of domain 1. Mg^2+^ is shown as a green sphere; dotted lines indicate hydrogen-bonding contacts; atoms that constitute ATP are colored cyan (carbon), red (oxygen), blue (nitrogen), and orange (phosphorus). (B) The ATP-binding capacity of mutant Rv2623 was compared to that of wild type protein following nucleotide extraction and HPLC. Data presented are derived from analysis of three independent protein preparations. ATP binding capacity is expressed as: [(the amount of ATP bound in mutant)/(the amount of ATP bound by wild type Rv2623) * 100]. (C) Thermal denaturation curves of two individual protein preparations of Rv2623_WT_ (WT-1, WT-2) as compared to Rv2623_G117A_ (G117A) and Rv2623_D15E_ (D15E). The data is expressed as the negative first-derivative of the fluorescence intensity as a function of temperature.

To examine the biochemical mechanisms responsible for Rv2623 function, we determined the crystal structure of wild-type Rv2623 at a resolution of 2.9 Å. The structure reveals a compact, 2-fold symmetric dimer. Each monomer is composed of tandem USP domains [residues 6–154 (domain 1), 155–294 (domain2)] that share 26% sequence identity and significant structural homology (residues 6–154 and 155–294 comprise domains 1 and 2, respectively; interdomain rms = 2.04 Å for 140 equivalent Cα's). Individual domains, which consist of a twisted, five-stranded, parallel β sheet flanked by four α helices, unite through an antiparallel, cross-strand (β5–β10) interaction that produces a central dyad axis between β5/β10 and a continuous, ten-stranded, mixed β sheet in the complete monomer. Each domain possesses a pair of conserved βαβ motifs (domain 1: β1-L1-α1- β2, β4-L2-α4-β5; domain 2: β6-L3-α5-β7, β9-L4-α8-β10) that encompass four loops (designated L1–L4) responsible for ATP recognition (Figure 6A and C). A “U-shaped” ATP molecule that lies within a cleft near the monomer surface is stabilized by 1) a cluster of hydrophobic residues (I14, V41, H42, V116/132/261/277/281, L136, A175) that forge the adenine/ribose-binding scaffold, 2) a pair of conserved L1/L3 aspartates (D15-L1/D167-L3), and 3) small phosphoryl/ribosyl-binding residues within the G-2X-G-9X-G (S/T) motifs that comprise L2/L4 (G120/265/267/268 and S131/276) (Figures 6A,C and 7A). Dimerization of Rv2623 occurs along a 2-fold axis orthogonal to the intramonomer dyad and juxtaposes ATP binding pockets from opposing monomers (Figure 6B).

Phylogenomic analysis places Rv2623 in a Uniprot/TrEMBL family (Q5YVE7) of 370 tandem-domain USPs, and a 113-member subfamily (N631) that consists almost exclusively of actinobacterial representatives (Text S1). Structure-based sequence alignments of both Rv2623 domains with the N631 consensus suggest that domain 2, which exhibits significantly higher conservation than domain 1 across global and ATP-binding subfamily consensus sequences, represents the ancestral domain among ATP-binding USPs with tandem-type architectures. Interestingly, the domain fold and interdomain organization observed for Rv2623 is broadly conserved: these features are shared among single domain USP structures, both monomeric and dimeric, that are presently represented within the PDB. As this manuscript was under preparation, a second, lower resolution (3.2 Å) crystal form of Rv2623 (PDB ID 2JAX) was released for public access. This structure is nearly identical to the present model as demonstrated by superposition over the ATP ligands and the monomeric and dimeric forms (rmsds are 0.57 and 0.81 for 258 and 517 matched CA's, respectively). The differences localize primarily to flexible loop regions (residues 44–58, 150–159) that, while disordered in 2JAX, are partially stabilized in the present structure by local crystal contacts.

To gain insight into the ATP-binding mode(s) exhibited by Rv2623, the structural features of the ATP-binding pocket of domains 1/2 were compared to the monomer fold of the representative ATP-binding USP, MJ0577 (PDBID 1MJH) [26]. Overlay of these structures reveals very considerable similarity for the residues that form the binding pockets and the associated ATP molecules, for which the triphosphoryl moieties assume virtually indistinguishable conformations. Relatively subtle structural and phylogenetic differences that exist between the ATP-binding pockets might nevertheless confer divergent binding and/or regulatory properties to the tandem domains.

### Generation and analysis of ATP-binding–deficient Rv2623 mutants

To explore the relationship between the putative ATP-dependent biochemical function of Rv2623 and the growth-regulating attribute of Rv2623, we engineered mutations within the L1 (D15E) and β4 (G117A) conserved residues that were predicted, on the basis of the crystal structure, to disrupt ATP recognition (Figure 7A). *In silico* replacement of the β4 G117 side chain hydrogen with a methyl group suggested that any residue larger than glycine at this position is likely to perturb both of the conserved loop regions in contact with the nucleotide. Similarly, extension of the D15 side chain to glutamate was also predicted to interfere with the ATP-binding conformation (Figure 7A). HPLC analysis of nucleotides extracted from Rv2623_D15E_ and Rv2623_G117A_ revealed that the mutant proteins are indeed deficient in ATP-binding, exhibiting ∼34% (p<0.001) and ∼29% (p = 0.0018) of the amount of ATP bound by wild-type Rv2623, respectively (Figure 7B). Likewise, following an overnight incubation with [α-^33^P] ATP at 4°C, the amount of protein-bound radioactivity, which represented a very small fraction of the total ATP binding sites, was significantly less for the mutant proteins than wild-type Rv2623 (data not shown). Importantly, thermal denaturation profiles of wild-type Rv2623, Rv2623_D15E_ and Rv2623_G117A_ demonstrated virtually identical Tm values, implying that the native Rv2623 fold was not destabilized by these mutations (Figure 7C). It is therefore likely that the D15E and G117A mutations produced local structural changes in the ATP binding loops that contributed directly to the reduced levels of bound ATP in comparing to wild-type Rv2623.

### The growth-regulating property of *M. tuberculosis* Rv2623 is dependent on its ability to bind ATP

**Figure 8:**
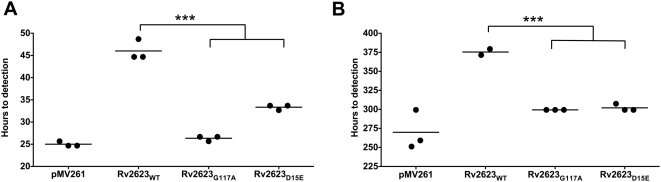
ATP binding by Rv2623 is required for its ability to attenuate growth. Growth of *M. smegmatis* (A) or *M. tuberculosis* (B) overexpressing wild type or mutant Rv2623 inoculated at 10^4^, 10^5^ CFU/ml into a BD BACTEC 9000MB system; the time to detection of triplicate cultures is indicated. While overexpression of wildtype Rv2623 attenuates mycobacterial growth, overexpression of either ATP-binding-deficient Rv2623 mutant (Rv2623_G117A_ or Rv2623_D15E_) significantly shifts the time to detection back towards that of the pMV261 vector control strain. ***p<0.001.

We next sought to probe the relationship between the nucleotide-binding capacity and growth regulation by this mycobacterial USP. Both the D15E and G117A mutant proteins were overexpressed in *M. smegmatis* mc^2^155 at levels equivalent to that of wild-type Rv2623 (Figure S4). Results of these studies demonstrated that while overexpression of wildtype Rv2623 retards the growth of the recipient strain relative to cells transformed with vector alone, growth of the strains overexpressing ATP-binding-deficient mutant Rv2623 are only minimally affected by overexpression as assessed by spotting serial dilutions of the cultures of the appropriate strains onto solid Middlebrook 7H10 agar (data not shown) as well as by monitoring the time to detection using the BD BACTEC 9000MB system (Figure 8A). The distinct effects exhibited by the wild type and the G117A and D15E mutants defective in ATP binding suggests a direct correlation between growth attenuation and ATP binding (Figure 7B). To examine whether the effects of overexpression of Rv2623 on *M. smegmatis* are operative in virulent *M. tuberculosis*, the growth kinetics of the Erdman strain overexpressing wildtype Rv2623, as well as the Rv2623_G117A_ and the Rv2623_D15E_ mutant proteins, were evaluated in vitro using the BACTEC 9000MB system (Figure 8B). As in the *M. smegmatis* studies, the results show that overexpression of Rv2623 in *M. tuberculosis* results in marked retardation of growth. Furthermore, this growth attenuation is not observed in *M. tuberculosis* strains overexpressing the G117A or the D15E mutant Rv2623 (Figure 8B). Taken together, these data strongly suggest that the ability of Rv2623 to regulate growth of *M. smegmatis* and *M. tuberculosis* is dependent on an ATP-dependent process.

## Discussion

Despite the significance of *M. tuberculosis* latency in pathogenesis, the mechanisms by which the tubercle bacillus establishes and maintains the latent state remain incompletely defined. Identification of *M. tuberculosis* genes that are induced by hypoxia and nitric oxide (NO) *in vitro* provides a framework for understanding the physiology of dormant bacilli [3],[4],[5]. These genes, referred to as the dormancy regulon, are transcriptionally regulated by the mycobacterial two-component system DosR-DosS under hypoxic conditions [4]. Indeed, it has been shown that both the cognate sensor histidine kinase DosS (a member of the dormancy regulon) as well as an “orphan” kinase, DosT, functioning as redox and hypoxia sensors, respectively; can regulate DosR activity, and that O_2_, NO, and CO can modulate the activity of these two kinases via interaction with a haem prosthetic group [28],[29],[30],[31],[32]. The biological significance of the dormancy regulon has been underscored by in vitro studies of *dosR* mutants of BCG and *M. tuberculosis*, which demonstrated the requirement of this transcription factor for survival under hypoxic conditions [3],[33]. Further, upregulation of the expression of certain dormancy regulon genes have been implicated in tuberculosis transmission as well as the virulence of the epidemiologically important W-Beijing lineage of *M. tuberculosis* [34],[35].

There are eight genes in the *M. tuberculosis* genome annotated to encode USP family proteins [7]. We studied the *M. tuberculosis* USP *rv2623* because it is one of the most highly induced genes in the dormancy regulon when bacilli are subjected to hypoxia and nitrosative stress [3],[4],[5],[36],[37]. More important, *rv2623* was also shown to be up-regulated when the tubercle bacillus is internalized by human and mouse macrophages [10],[38] as well as in the lungs of mice with persistent *M. tuberculosis* infection [11]. These latter observations suggest that the induction of *rv2623* may have biological relevance. The precise mechanisms by which Rv2623 expression is regulated remain to be defined. Recent transcriptional analysis of Rv2623, while confirming the essentiality of the two 18 bp palindromic DosR-binding motifs that are present in the promoter region of this gene [38] for induction of Rv2623 under low oxygen conditions, also demonstrated the presence of additional regulatory elements within the *rv2623* 5′-untranslated region [18]. These results suggest that the regulation of Rv2623 is likely complex.

The *M. tuberculosis* dormancy response features a dramatic decrease in metabolic activity, resulting in a rapid decrease in bacterial replication [39]. Therefore, it is possible that deficiency in certain members of the dormancy regulon could result in inability of the tubercle bacillus to enter a latent state in the infected host, leading to unrestrained growth and thus, hypervirulence. Indeed, specific members of the *M. tuberculosis* dormancy regulon whose insufficiency results in a hypervirulence phenotype have been reported [40],[41]. In certain experimental tuberculosis animal models, DosR deficiency has been associated with a hypervirulence phenotype [41]. However, DosR deficiency has also been reported to have no effect on *M. tuberculosis* virulence or to lead to an attenuated phenotype [42],[43]. The discrepancies regarding *M. tuberculosis* virulence in these DosR studies are unclear, but could be due to differences in experimental systems employed. Insufficiency of the chaperone-like α-crystallin encoded by *M. tuberculosis hspX* (*acr*) has also been shown to be associated with hypervirulence in a BALB/c mouse model of tuberculosis [40].

In the present study, an *rv2623* knockout mutant of virulent *M. tuberculosis* Erdman fails to establish a chronic persistent infection, displaying a hypervirulent phenotype in susceptible hosts, as assessed by lung bacterial burden, histopathology, and mortality. Results of the complementation studies indicate that the phenotype is Rv2623-specific. This growth-regulating phenomenon is echoed by the observation that ectopic overexpression of Rv2623 results in attenuation of mycobacterial growth. Together, these data strongly suggest that the *M. tuberculosis* USP Rv2623 has the ability to regulate growth *in vitro* and *in vivo*, and is required for the establishment of a persistent infection. Intriguingly, ectopic overexpression of HspX by the same means employed by our study also resulted in an attenuated growth phenotype compared with LacZ-overexpressing controls [44], suggesting that these two tightly co-regulated “stress” proteins might have similar growth-regulatory roles during dormancy.

Bioinformatic and experimental evidence suggest that nucleotide-binding capacity represents a discriminating biochemical feature that facilitates USP protein classification. Putative functional differences between USPs are implied by their assignment to two subclasses: one whose members do not bind ATP and another whose constituents bind and hydrolyze adenine nucleotide substrates [8],[26],[27],[45],[46]. A structural comparison between the prototypic members of the two subclasses, the non-ATP-binding UspA homolog (*H. influenzae*, PDB ID 1JMV) and the ATP-binding USP, MJ0577 (*M. jannaschii*, PDB ID 1MJH) revealed that while both proteins exhibit a similar fold, conserved glycine residues within the ATP-binding loop of the latter are substituted with bulky amino acids that preclude ATP recognition in the former [26],[27]. The unique nucleotide-binding pocket of this protein family is structurally distinct from those commonly encountered in ATP-binding proteins [26],[47],[48]. Specific roles for USP family proteins are just beginning to be characterized, and early functional classifications have been informed by ATP-binding capacity [9]. While the non-ATP-binding UspA homologs appear to play diverse roles in promoting survival under a variety of environmental insults [15],[49],[50],[51], the function(s) of ATP-binding type USPs remain unclear [9]. Based on *in silico* analyses, Florczyk et al. classified Rv2623 as belonging to a novel class of ATPases [52], although formal evidence for ATP binding by this protein has not been reported.

This study has provided substantial biochemical and structural evidence that *M. tuberculosis* Rv2623 is a *bona fide* nucleotide-binding USP: i) *E. coli-*expressed His_6_-Rv2623 co-purifies with tightly bound ATP and ADP; ii) analysis of the 2.9 Å -resolution Rv2623 crystal structure, the first molecular model of a tandem-type USP, reveals four ATP-bound nucleotide-binding pockets; and iii) point mutations (D15E, G117A) within the conserved L1 (D15E) and β4 (G117A) regions of the structure, which were predicted to disrupt nucleotide-binding, yielded mutant proteins with attenuated ATP-binding capacity. Furthermore, given that the attenuated growth phenotype caused by overexpression of Rv2623 could be abrogated by mutations that interfere with the binding of this protein to its nucleotide substrate, it is likely that the mycobacterial growth-regulatory faculty of Rv2623 is mediated by an ATP-dependent function.

In summary, the results of the present study have revealed that the *M. tuberculosis* USP Rv2623 has the ability to regulate mycobacterial growth, as evident by the *in vivo* hypervirulence phenotype of Δ*rv2623*, which fails to establish a persistent infection in susceptible hosts, as well as the growth attenuation observed in mycobacteria overexpressing this USP. Thus, *M. tuberculosis* Rv2623 may serve the function of promoting mycobacterial transition into latency. The latent state allows persistence in infected individuals of tubercle bacilli that can reactivate to cause active disease and to disseminate when the immune status of the host is compromised. As a result, Rv2623 may contribute significantly to the propagation of the tubercle bacillus in the human host and the difficulties in eradicating tuberculosis. Mechanistically, results of the mutagenesis studies have shown that Rv2623 regulates growth through ATP-dependent function. Clearly, much remains to be learned regarding how the ATP-dependent function of Rv2623 contributes to growth regulation. It has been proposed that a nucleotide-binding USP from *M. jannaschii*, MJ0577, whose ability to hydrolyze ATP is dependent on interaction with factor(s) present in the cell extract of this hyperthermophile [26], functions as a molecular switch much like the Ras protein family, whose GTP hydrolysis ability is modulated by interaction with a number of regulatory proteins [53],[54],[55]. The fact that *E. coli-*expressed Rv2623 co-purifies with ADP as well as ATP suggests the possibility that this mycobacterial USP, like MJ0577, is capable of ATP hydrolysis. It is therefore conceivable that *M. tuberculosis* Rv2623, as a component of the yet-to-be defined dormancy signaling pathway(s), functions as a molecular switch by virtue of its ATP-binding and putative ATP-hydrolyzing properties, to mediate the establishment of tuberculous latency. Experiments designed to investigate the potential ATP-hydrolyzing activity of Rv2623 are currently underway. Recent identification of the DosR-dependent dormancy regulon [3],[4],[5]; the DosR-independent enduring hypoxic response, which involves over 200 mycobacterial genes, including those known to regulate bacteriostasis [42]; and the demonstration that *M. tuberculosis* redox and hypoxia sensors can interact with multiple ligands that differentially modulate the activity of these important kinases [28],[29],[30],[31],[32], predict a complex regulatory network for tuberculous latency. Elucidation of how ATP-binding and, potentially, the hydrolysis of ATP by Rv2623 regulate *M. tuberculosis* dormancy-signaling pathways will likely illuminate the mechanisms by which the tubercle bacillus establishes persistence.

## Materials and Methods

### Bacterial strains and culture conditions

Liquid cultures of *M. tuberculosis* and *M. smegmatis* strains were grown in Middlebrook 7H9 medium (Becton Dickinson, Sparks, MD) supplemented with 0.2% glycerol (Sigma, St. Louis, MO), 0.05% Tween 80 (Sigma, St. Louis, MO), and 10% oleic acid-albumin-dextrose-catalase (OADC) enrichment media (Becton Dickinson, Sparks, MD). For the determination of the number of colony forming units (CFU) and examination of growth on solid media, Middlebrook 7H10 agar medium (Becton Dickinson) supplemented with 0.5% glycerol and 10% OADC was used. The Δ*rv2623* mutant strain was maintained in media supplemented with 50 µg/ml hygromycin B (Roche) and cultures of complemented, Rv2623-overexpressing strains contained kanamycin (40 µg/ml). Growth was also examined in minimal Sauton's medium (4 g asparagine, 2 g sodium citrate, 0.5 g K_2_HPO_4_·3H_2_O, 0.5 g MgSO_4_·7H_2_O, 0.05 g ferric ammonium citrate, 60 g glycerol in 1 L of H_2_O supplemented with 0.05% Tween80 and antibiotics as required). For some experiments, growth was monitored by the BD BACTEC 9000 system (Becton Dicinson). Stationary phase *M. tuberculosis* or *M. smegmatis* cultures were inoculated in triplicates (10^5^ or 10^4^ CFU) into vials of liquid medium containing a sensor compound that fluoresces upon depletion of oxygen as a result of bacterial growth. The time to detection reflects the rate of bacterial growth.

### Generation of Δ*rv2623* mutant strain, complemented and overexpression strains

Replacement of genomic *rv2623* was performed by allelic exchange using a specialized transducing phage delivery system as previously described [12]. Transformants were analyzed by PCR and Southern blot to confirm replacement of *rv2623* with a hygromycin cassette, yielding Δ*rv2623*. A complemented strain was generated as described previously [56] by transformation of Δ*rv2623* with a plasmid vector that integrates at the *attB* site and bears the *rv2623* coding sequence under transcriptional control of the constitutive *hps60* promoter or the endogenous *rv2623* promoter [18], yielding Δ*rv2623 attB*::P_hsp60_ Rv2623 and Δ*rv2623 attB*::P_rv2623_ Rv2623; respectively. The *M.tuberculosis hsp60* promoter fusion was also used to overexpress Rv2623 via subcloning of this region into pMV261, a non-integrating variant of pMV361 to yield pMV261::*rv2623*, which is self-replicating at 3–5 copies/cell [57] (Text S1).

### Response of Δ*rv2623* mutant to stress

Log phase cultures (OD_600_ = 0.8–1.0) of Erdman and Δ*rv2623* were diluted 1∶10 into Sauton's media containing various stress-inducing chemical agents (phenazine methosulfate, streptonigrin and mitomycin C) at the indicated concentrations or for acid stress into 7H9+10% OADC+0.05% Tween 80 (pH = 4.0) and grown at 37°C for several days. Growth was monitored by OD_600_. Survival of these strains following heat shock was compared after a shift of log phase cultures from 37°C to 53°C and determining the number of CFU/ml at various time points thereafter. For irradiation with UV light, cells were plated onto solid 7H10 agar supplemented with 0.05% Tween80 and exposed to increasing amounts of UV energy (UV Stratalinker 1800, Stratagene). Surviving cells were enumerated and the data are expressed as percent survival as compared to unexposed controls. In addition, cells were treated with the indicated concentrations of mitomycin C for a period of 1 hour followed by determination of surviving CFU/ml. See Figure S2 for details.

### Animal infections

Outbred Hartley guinea pigs (∼500 g body weight) (Charles River Laboratories, North Wilmington, MA) were given a low dose of *M. tuberculosis* using a Madison chamber aerosol generation device calibrated to deliver ∼30 CFU [58]. Guinea pigs were sacrificed (n = 5) at 20, 40, and 60 days post infection for histological analysis and determination of organ bacterial burden. Histological analysis of infected tissues was performed by scoring individual tissue sections based on criteria described in Protocol S1.

For the murine tuberculosis model, six-to-eight-week-old mice (Jackson Laboratories, Bar Harbor, Maine) were infected with *M. tuberculosis* via aerosol (In-Tox Products, Albuquerque, NM) as previously described [19] with ∼100 CFU (C57BL/6) or 750–1000 CFU (C3H/HeJ, C3HeB/FeJ). For CFU determination, mice were sacrificed (n = 3) at the times indicated and portions of the lung, liver and spleen were homogenized in PBS+0.05% Tween 80, diluted, and plated onto solid 7H10 media.

### Cloning, expression and purification of Rv2623

The coding sequence of *rv2623* was PCR-amplified from *M. tuberculosis* Erdman genomic DNA and subcloned into the expression vector pQE80L (Qiagen, Inc.), which encodes an N-terminal His_6_-tag, producing the plasmid pQE-*rv2623* (Text S1). Expression was carried out following isopropyl beta-D-thiogalactoside (IPTG) induction of BL21 *E. coli* transformed with pQE-*rv2623*. His_6_-Rv2623 was then affinity-purified to homogeneity from BL21 cell lysates using Ni-NTA agarose (Qiagen, Inc.) according to the manufacturer's instructions. For crystallization, purified Rv2623 was concentrated to 12 mg/ml using a 10 kDa Molecular Weight Cut Off (MWCO) centrifugal filter (Amicon), and frozen at −80°C.

### Gel filtration chromatography

A Superdex 200 10/300 GL column (GE Healthcare Life Sciences) was equilibrated with Rv2623 dialysis buffer (Text S1) and calibrated using the following molecular mass standards: aldolase (158 kDa), bovine serum albumin (67 kDa), ovalbumin (43 kDa), chymotrypsinogen A (25 kDa) as described in the Amersham Pharmacia technical notes (GE Healthcare Life Sciences). The flow rate was set to 0.15 ml/min and elution of the protein was monitored at 280 nm.

### High performance liquid chromatography (HPLC)

Nucleotides were extracted from purified Rv2623 by boiling. Samples were then loaded onto an analytical, anion-exchange HPLC column (AX300, Eprogen Inc. or Mono Q HR 5/5, GE Healthcare). Samples were eluted isocratically using NaH_2_PO_4_, pH 5.5 (AX300) or using a ammonium phosphate pH 7.0 (0.02–1.0 M) step gradient (Mono Q) (Text S1). Nucleotides were identified on the basis of retention time relative to nucleotide standards, and quantified by peak area.

### Stoichiometry of nucleotide binding

Following Ni affinity chromatography, His_6_-Rv2623 samples for stoichiometry measurements (500 µl, 8–120 µM protein) were attained by subjecting the Ni-purified fractions to rapid desalting over a HiPrep Desalting 26/10 column (GE Healthcare) equilibrated with 50 mM NaCl, 2 mM MgCl_2_, 10% glycerol, 20 mM HEPES pH 8.0 and/or an additional purification using a MonoQ 10/100 GL column (GE Healthcare) equilibrated with the desalting buffer and eluted with a linear salt gradient ranging from 50 mM to 250 mM NaCl. Amicon centrifugal filtration concentrators (MWCO = 30 kDa) were employed for final concentration steps prior to analysis. Bound nucleotide was then released by boiling and quantified by HPLC (under the Mono Q HR 5/5 conditions) according to the methodology described above and Text S1. The heat-precipitated protein was subsequently reconstituted in 6 M guanidine HCl and its concentration was determined by spectrophotometric measurement of the protein peak at 280 nM and a molar extinction coefficient for Rv2623 at 280 nm (54,640/Mcm). This extinction coefficient was determined using a 6 M GuHCl-reconstituted (nucleotide-free) sample of Rv2623 that had been subject to quantitative amino acid analysis at the Yale Keck Facility. The nucleotide binding stoichiometry was calculated as the molar ratio of the released nucleotide to protein.

### Site-directed mutagenesis of Rv2623

Single amino acid substitutions were incorporated into the *rv2623* coding region contained in appropriate expression vectors by mismatched PCR priming (Text S1). Individual PCR reactions were performed using either pMV261::*rv2623* or pQE-*rv2623* plasmid templates for mycobacterial overexpression and protein purification, respectively. Then the pMV261:: *rv2623* mutants expression vector DNA was used to transformed into *M.smegamati*s mc^2^155 and the DNA of pQE-*rv2623* mutants was transformed into *E.coli* BL21(DE3). Thermal denaturation curves were determined for purified wild type and mutant Rv2623 using an IQ5 Real Time PCR Detection System (Bio-Rad) following incubation with SYPRO Orange protein gel stain (Invitrogen) (Text S1).

### Crystallization and data collection

ATP and MgCl_2_ were added to final concentrations of 0.8 mM and 1.8 mM, respectively, in the protein sample prior to crystallization by sitting-drop vapor-diffusion at 4°C (Text S1). Diffraction data were collected at the National Synchrotron Light Source beamline X29A on an ADSC Q315 detector through the Macromolecular Crystallography Research Resource (PXRR) mail-in crystallography program. Data processing and scaling was performed with the HKL2000 suite.

### Structure determination and refinement

The structure *of M. tuberculosis* Rv2623, which contains two USP domains in tandem, and whose first domain shares 25% sequence identity with the USP *M. tuberculosis* Rv1636, was solved using the molecular replacement method and a CHAINSAW-generated search model consisting of the Rv1636 dimer (PDB ID 1TQ8 chains A, B), using a 2.9 Å, C222_1_ dataset (a = 173, b = 241.5, c = 241.7). A starting polyalanine model (R/R_free_ = .56/.57) of four dimers was subject to four refinement cycles, each consisting of multi-domain rigid-body refinement in Molrep, a single cycle of restrained MLF refinement in Refmac5 (to obtain input FOMs for DM), 20 cycles of phase extension in DM (as above), and manual rebuilding of the polyalanine backbone in Coot. As R-factors converged (R/R_free_ = .40/.42), ∼80% of the side chains were positioned and the Rv2623 dimers were further rebuilt and refined (R/R_free_ = .31/.33) in CNS using high NCS restraint weights (400 kcal/mol) with rigid-body, energy minimization, grouped isotropic B factor, and simulated annealing refinement protocols. ATP and Mg^2+^ were built within composite omit density (calculated in CNS) during the final rebuilding/refinement cycles conducted with relaxed NCS restraints in Arp-waters and Refmac5, yielding final R/R_free_ = 24.5/26.5. SigmaA-weighted difference maps calculated with the refined model reveal weak, fragmented density for a pseudotranslated copy of the Rv2623 dimer whose corresponding NCS translational vector (uvw = .500, .012, .494) appears in the native patterson at 7.7% of the origin peak height. Data collection and refinement statistics are summarized in Protocol S1. The coordinates of *M. tuberculosis* Rv2623 have been submitted to the protein databank (PBDID 3CIS).

## Supporting Information

Figure S1 Survival curve of C3HeB/FeJ mice infected with wildtype of Δ*rv2623M. tuberculosis* Erdman. C3HeB/FeJ mice were infected via aerosol with a high dose 750–1000 CFU of either Erdman or Δ*rv2623M. tuberculosis* (n = 6). The median survival times for Erdman- and Δ*rv2623*-infected mice are 56 and 27 days post infection, respectively (p = 0.0037). Statistical significance was determined using GraphPad Prism version 4.0c for Mac, GraphPad Software, San Diego California, USA, www.graphpad.com; ** indicates p<0.01.
(2.62 MB TIF)

Figure S2 Characterization of the response of Δ*rv2623* to environmental stress. Optical density(OD_600_) was used to monitor the growth of Erdman (solid line) and Δ*rv2623* (dashed line) cultures in the presence of (A) superoxide anion (O_2_^−^) following 1∶10 dilution of log-phase culture into fresh Sauton's media containing 10 µM (triangles) or 20 µM (circles) phenazine methosulfate (PMS) or (B) the Fe^3+^-dependent quinone antibiotic streptonigrin at concentrations of 0.01 µM (triangles) or 0.1 µM (circles). For (A,B): Untreated control cultures are represented by square symbols. (C) Sensitivity to ultraviolet irradiation was examined by exposing cells on solid media to the indicated energies and determining the number of surviving bacilli. Data are expressed as a percent survival relative to unexposed control plates for each strain. (D) The number of surviving CFU/ml was determined following treatment of log phase culture with increasing amounts of mitomycin C for 1 hour at 37°C. (E) Similarly, the number of surviving bacilli was determined following incubation at 53°C for the indicated times. (F) Log phase cultures were diluted 1∶10 into media at pH 7.0 or pH 4.0 and grown for several days and the number of surviving CFU/ml was monitored. Error bars represent the standard error of the means; each is a combination of at least two independent experiments performed in triplicate.
(15.14 MB TIF)

Figure S3 Purification and gel filtration profile of His_6_-Rv2623. (A) Expression and purification of His_6_-Rv2623. Lane 1: Uninduced pQE-*rv2623E.coli* cell lysate carrying expression construct pQE-*rv2623*; Lane 2: Induced pQE-*rv2623E.coli* cell lysate; Lane 3 and Lane 4: Purified His_6_-Rv2623: 0.3 mg and 3 mg, respectively; Lane 5: Western blot probed with anti-RGS-His antibody. Arrow indicates His_6_-Rv2623 (32 kDa). (B) Gel filtration profile of purified His_6_-Rv2623 eluted from a Superdex 200 10/300 GL column. Molecular weight (61 kDa) was determined based on the specific retention time corresponding to the His_6_-Rv2623 peak compared to molecular weight standards. The predicted molecular weight of Rv2623 is 32 kDa and purified protein is representative of a dimer of Rv2623.
(5.69 MB TIF)

Figure S4 Relative expression level of wildtype(WT) and mutant Rv2623 in *M. smegmatis*. Strains of *M. smegmatis* that carried pMV261::*rv2623*_WT_, pMV261::*rv2623*_G117A_, and pMV261::*rv2623*_D15E_ overexpression plasmids were grown to late log phase in 7H9 media supplemented with kanamycin (40 µg/ml). The OD_600_ of the cultures was measured and used to estimate the number of CFU/ml (conversion factor: OD_600_ 1 = 3×10^8^ CFU/ml). An equivalent number of cells for each strain were boiled for 5 minutes in denaturing sample loading buffer and separated by SDS-PAGE. (A) Western blotting techniques were used to detect Rv2623 in the total protein extract from these strains using monoclonal anti-Rv2623. (B) The immunoblot was scanned and analyzed with ImageQuant densitometry software (Molecular Dynamics). As different amounts of cells were boiled in different experiments, the relative intensity of the bands is expressed as a percent of the signal corresponding to Rv2623_WT_ overexpression so that multiple experiments could be combined. The error bars represent the standard deviation and the percent of wild type Rv2623 expression is indicated above the bars. The data indicates that mutant Rv2623 overexpressed at similar levels compared to WT Rv2623.
(4.75 MB TIF)

Protocol S1 Semiquantitative scoring system for immunopathology of guinea pig tissues; and Crystallography: Summary of data collection and refinement statistics.
(2.82 MB PDF)

Table S1 Gene list and accession numbers.
(0.03 MB DOC)

Text S1 Supporting materials and methods.
(0.03 MB DOC)

## References

[1] WHO (2007). Global Tuberculosis Control: Surveillance, Planning, and Financing (2007.378).

[2] Flynn JL, Chan J (2001). Immunology of tuberculosis.. Annu Rev Immunol.

[3] Voskuil MI, Schnappinger D, Visconti KC, Harrell MI, Dolganov GM (2003). Inhibition of respiration by nitric oxide induces a Mycobacterium tuberculosis dormancy program.. J Exp Med.

[4] Park HD, Guinn KM, Harrell MI, Liao R, Voskuil MI (2003). Rv3133c/dosR is a transcription factor that mediates the hypoxic response of Mycobacterium tuberculosis.. Mol Microbiol.

[5] Ohno H, Zhu G, Mohan VP, Chu D, Kohno S (2003). The effects of reactive nitrogen intermediates on gene expression in Mycobacterium tuberculosis.. Cell Microbiol.

[6] Nystrom T, Neidhardt FC (1992). Cloning, mapping and nucleotide sequencing of a gene encoding a universal stress protein in Escherichia coli.. Mol Microbiol.

[7] O'Toole R, Williams HD (2003). Universal stress proteins and Mycobacterium tuberculosis.. Res Microbiol.

[8] Kvint K, Nachin L, Diez A, Nystrom T (2003). The bacterial universal stress protein: function and regulation.. Curr Opin Microbiol.

[9] Nachin L, Nannmark U, Nystrom T (2005). Differential roles of the universal stress proteins of Escherichia coli in oxidative stress resistance, adhesion, and motility.. J Bacteriol.

[10] Monahan IM, Betts J, Banerjee DK, Butcher PD (2001). Differential expression of mycobacterial proteins following phagocytosis by macrophages.. Microbiology.

[11] Shi L, Jung YJ, Tyagi S, Gennaro ML, North RJ (2003). Expression of Th1-mediated immunity in mouse lungs induces a Mycobacterium tuberculosis transcription pattern characteristic of nonreplicating persistence.. Proc Natl Acad Sci U S A.

[12] Bardarov S, Bardarov S, Pavelka MS, Sambandamurthy V, Larsen M (2002). Specialized transduction: an efficient method for generating marked and unmarked targeted gene disruptions in Mycobacterium tuberculosis, M. bovis BCG and M. smegmatis.. Microbiology.

[13] Bochkareva ES, Girshovich AS, Bibi E (2002). Identification and characterization of the Escherichia coli stress protein UP12, a putative in vivo substrate of GroEL.. Eur J Biochem.

[14] Nystrom T, Neidhardt FC (1994). Expression and role of the universal stress protein, UspA, of Escherichia coli during growth arrest.. Mol Microbiol.

[15] Gustavsson N, Diez A, Nystrom T (2002). The universal stress protein paralogues of Escherichia coli are co-ordinately regulated and co-operate in the defence against DNA damage.. Mol Microbiol.

[16] Snapper SB, Melton RE, Mustafa S, Kieser T, Jacobs WR (1990). Isolation and characterization of efficient plasmid transformation mutants of Mycobacterium smegmatis.. Mol Microbiol.

[17] Liu WT, Karavolos MH, Bulmer DM, Allaoui A, Hormaeche RD (2007). Role of the universal stress protein UspA of Salmonella in growth arrest, stress and virulence.. Microb Pathog.

[18] Vasudeva-Rao HM, McDonough KA (2008). Expression of the Mycobacterium tuberculosis acr-coregulated genes (ACGs) from the DevR (DosR) regulon is controlled by multiple levels of regulation.. Infect Immun.

[19] Scanga CA, Mohan VP, Tanaka K, Alland D, Flynn JL (2001). The inducible nitric oxide synthase locus confers protection against aerogenic challenge of both clinical and laboratory strains of Mycobacterium tuberculosis in mice.. Infect Immun.

[20] Baldwin SL, D'Souza C, Roberts AD, Kelly BP, Frank AA (1998). Evaluation of new vaccines in the mouse and guinea pig model of tuberculosis.. Infect Immun.

[21] McMurray DN (2001). Disease model: pulmonary tuberculosis.. Trends Mol Med.

[22] Scott HM, Flynn JL (2002). Mycobacterium tuberculosis in chemokine receptor 2-deficient mice: influence of dose on disease progression.. Infect Immun.

[23] Tsai MC, Chakravarty S, Zhu G, Xu J, Tanaka K (2006). Characterization of the tuberculous granuloma in murine and human lungs: cellular composition and relative tissue oxygen tension.. Cell Microbiol.

[24] Chackerian AA, Perera TV, Behar SM (2001). Gamma interferon-producing CD4+ T lymphocytes in the lung correlate with resistance to infection with Mycobacterium tuberculosis.. Infect Immun.

[25] Schnappinger D, Ehrt S, Voskuil MI, Liu Y, Mangan JA (2003). Transcriptional Adaptation of Mycobacterium tuberculosis within Macrophages: Insights into the Phagosomal Environment.. J Exp Med.

[26] Zarembinski TI, Hung LW, Mueller-Dieckmann HJ, Kim KK, Yokota H (1998). Structure-based assignment of the biochemical function of a hypothetical protein: a test case of structural genomics.. Proc Natl Acad Sci U S A.

[27] Sousa MC, McKay DB (2001). Structure of the universal stress protein of Haemophilus influenzae.. Structure.

[28] Roberts DM, Liao RP, Wisedchaisri G, Hol WG, Sherman DR (2004). Two sensor kinases contribute to the hypoxic response of Mycobacterium tuberculosis.. J Biol Chem.

[29] Kumar A, Toledo JC, Patel RP, Lancaster JR, Steyn AJ (2007). Mycobacterium tuberculosis DosS is a redox sensor and DosT is a hypoxia sensor.. Proc Natl Acad Sci U S A.

[30] Sousa EH, Tuckerman JR, Gonzalez G, Gilles-Gonzalez MA (2007). DosT and DevS are oxygen-switched kinases in Mycobacterium tuberculosis.. Protein Sci.

[31] Sardiwal S, Kendall SL, Movahedzadeh F, Rison SC, Stoker NG (2005). A GAF domain in the hypoxia/NO-inducible Mycobacterium tuberculosis DosS protein binds haem.. J Mol Biol.

[32] Saini DK, Malhotra V, Tyagi JS (2004). Cross talk between DevS sensor kinase homologue, Rv2027c, and DevR response regulator of Mycobacterium tuberculosis.. FEBS Lett.

[33] Boon C, Dick T (2002). Mycobacterium bovis BCG response regulator essential for hypoxic dormancy.. J Bacteriol.

[34] Reed MB, Gagneux S, Deriemer K, Small PM, Barry CE (2007). The W-Beijing lineage of Mycobacterium tuberculosis overproduces triglycerides and has the DosR dormancy regulon constitutively upregulated.. J Bacteriol.

[35] Garton NJ, Waddell SJ, Sherratt AL, Lee SM, Smith RJ (2008). Cytological and transcript analyses reveal fat and lazy persister-like bacilli in tuberculous sputum.. PLoS Med.

[36] Boon C, Li R, Qi R, Dick T (2001). Proteins of Mycobacterium bovis BCG induced in the Wayne dormancy model.. J Bacteriol.

[37] Rosenkrands I, Slayden RA, Crawford J, Aagaard C, Barry CE (2002). Hypoxic response of Mycobacterium tuberculosis studied by metabolic labeling and proteome analysis of cellular and extracellular proteins.. J Bacteriol.

[38] Florczyk MA, McCue LA, Purkayastha A, Currenti E, Wolin MJ (2003). A family of acr-coregulated Mycobacterium tuberculosis genes shares a common DNA motif and requires Rv3133c (dosR or devR) for expression.. Infect Immun.

[39] Wayne LG, Sohaskey CD (2001). Nonreplicating persistence of mycobacterium tuberculosis.. Annu Rev Microbiol.

[40] Hu Y, Movahedzadeh F, Stoker NG, Coates AR (2006). Deletion of the Mycobacterium tuberculosis alpha-crystallin-like hspX gene causes increased bacterial growth in vivo.. Infect Immun.

[41] Parish T, Smith DA, Kendall S, Casali N, Bancroft GJ (2003). Deletion of two-component regulatory systems increases the virulence of Mycobacterium tuberculosis.. Infect Immun.

[42] Rustad TR, Harrell MI, Liao R, Sherman DR (2008). The Enduring Hypoxic Response of Mycobacterium tuberculosis.. PLoS ONE.

[43] Malhotra V, Sharma D, Ramanathan VD, Shakila H, Saini DK (2004). Disruption of response regulator gene, devR, leads to attenuation in virulence of Mycobacterium tuberculosis.. FEMS Microbiol Lett.

[44] Yuan Y, Crane DD, Barry CE (1996). Stationary phase-associated protein expression in Mycobacterium tuberculosis: function of the mycobacterial alpha-crystallin homolog.. J Bacteriol.

[45] Weber A, Jung K (2006). Biochemical properties of UspG, a universal stress protein of Escherichia coli.. Biochemistry.

[46] Saveanu C, Miron S, Borza T, Craescu CT, Labesse G (2002). Structural and nucleotide-binding properties of YajQ and YnaF, two Escherichia coli proteins of unknown function.. Protein Sci.

[47] Walker JESM, Runswick MJ, Gay NJ (1982). Distantly related sequences in the alpha- and beta-subunits of ATP synthase, myosin, kinases and other ATP-requiring enzymes and a common nucleotide binding fold.. EMBO J.

[48] Traut TW (1994). The functions and consensus motifs of nine types of peptide segments that form different types of nucleotide-binding sites.. Eur J Biochem.

[49] Boes N, Schreiber K, Hartig E, Jaensch L, Schobert M (2006). The Pseudomonas aeruginosa Universal Stress Protein PA4352 Is Essential for Surviving Anaerobic Energy Stress.. J Bacteriol.

[50] Chen W, Honma K, Sharma A, Kuramitsu HK (2006). A universal stress protein of Porphyromonas gingivalis is involved in stress responses and biofilm formation.. FEMS Microbiol Lett.

[51] Mangalappalli-Illathu AK, Korber DR (2006). Adaptive Resistance and Differential Protein Expression of Salmonella enterica Serovar Enteritidis Biofilms Exposed to Benzalkonium Chloride.. Antimicrob Agents Chemother.

[52] Florczyk MA, McCue LA, Stack RF, Hauer CR, McDonough KA (2001). Identification and characterization of mycobacterial proteins differentially expressed under standing and shaking culture conditions, including Rv2623 from a novel class of putative ATP-binding proteins.. Infect Immun.

[53] Siderovski DP, Willard FS (2005). The GAPs, GEFs, and GDIs of heterotrimeric G-protein alpha subunits.. Int J Biol Sci.

[54] Quilliam LA, Rebhun JF, Castro AF (2002). A growing family of guanine nucleotide exchange factors is responsible for activation of Ras-family GTPases.. Prog Nucleic Acid Res Mol Biol.

[55] Walker SA, Lockyer PJ, Cullen PJ (2003). The Ras binary switch: an ideal processor for decoding complex Ca2+ signals?. Biochem Soc Trans.

[56] Tufariello JM, Jacobs WR, Chan J (2004). Individual Mycobacterium tuberculosis resuscitation-promoting factor homologues are dispensable for growth in vitro and in vivo.. Infect Immun.

[57] Stover CK, de la Cruz VF, Fuerst TR, Burlein JE, Benson LA (1991). New use of BCG for recombinant vaccines.. Nature.

[58] Lasco TM, Turner OC, Cassone L, Sugawara I, Yamada H (2004). Rapid accumulation of eosinophils in lung lesions in guinea pigs infected with Mycobacterium tuberculosis.. Infect Immun.

