# Correction: *Mycobacterium tuberculosis* Universal Stress Protein Rv2623 Regulates Bacillary Growth by ATP-Binding: Requirement for Establishing Chronic Persistent Infection

**DOI:** 10.1371/annotation/2b1a4b06-9558-448b-a8e8-5e2d407816a0

**Published:** 2009-09-16

**Authors:** Joshua E. Drumm, Kaixia Mi, Patrick Bilder, Meihao Sun, Jihyeon Lim, Helle Bielefeldt-Ohmann, Randall Basaraba, Melvin So, Guofeng Zhu, JoAnn M. Tufariello, Angelo A. Izzo, Ian M. Orme, Steve C. Almo, Thomas S. Leyh, John Chan

The first three authors, Joshua E. Drumm, Kaixia Mi, and Patrick Bilder, should be noted as contributing equally to this work. 

