# Supplementary figures and images for: Mycobacterium tuberculosis Universal Stress Protein Rv2623 Regulates Bacillary Growth by ATP-Binding: Requirement for Establishing Chronic Persistent Infection
Source: PLoS Pathog. 2009 May 29;5(5):e1000460. doi: 10.1371/journal.ppat.1000460 (PMC2682197; doi:10.1371/journal.ppat.1000460)

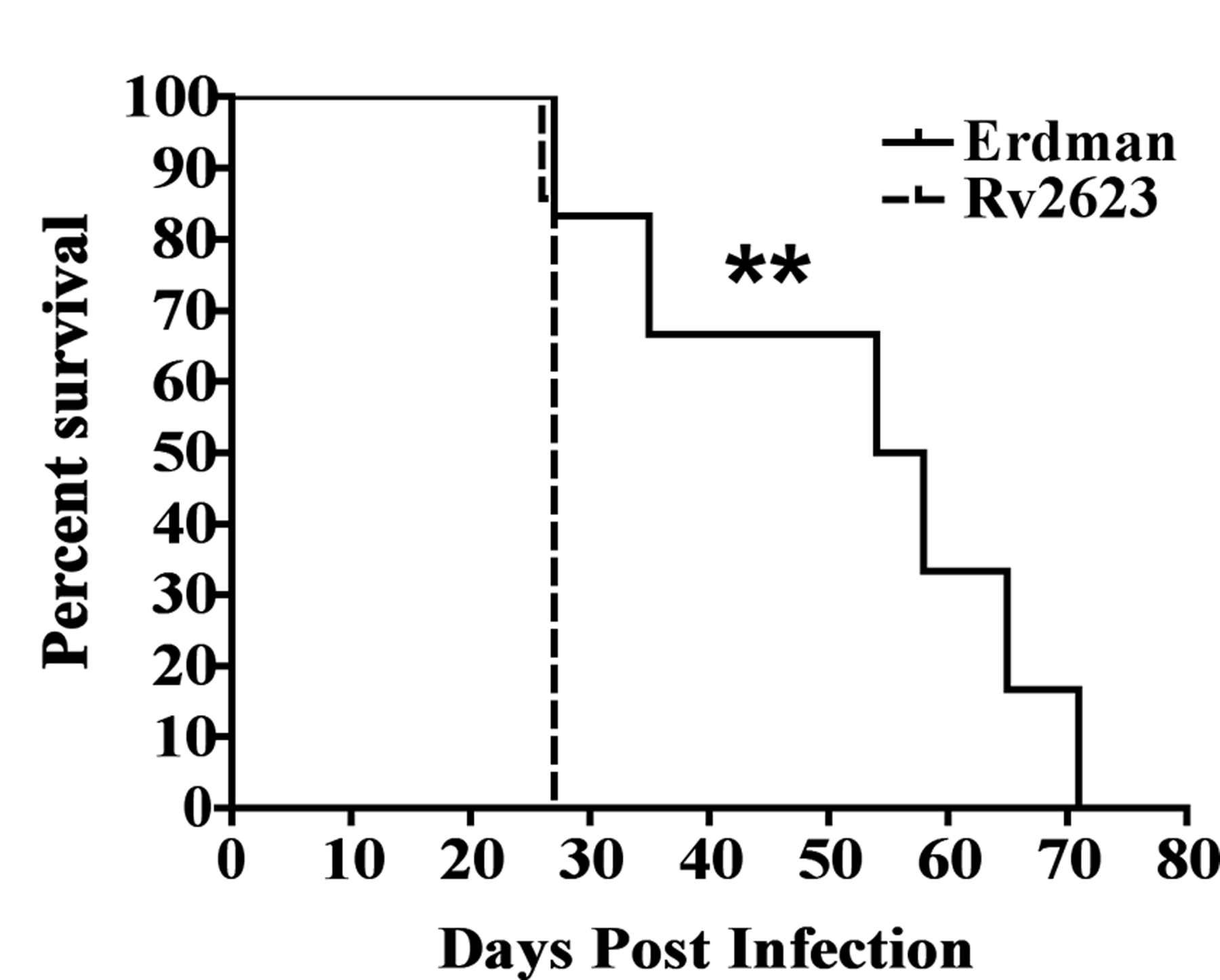

Supplement: Figure S1 — Survival curve of C3HeB/FeJ mice infected with wildtype of Δrv2623M. tuberculosis Erdman. C3HeB/FeJ mice were infected via aerosol with a high dose 750–1000 CFU of either Erdman or Δrv2623M. tuberculosis (n = 6). The median survival times for Erdman- and Δrv2623-infected mice are 56 and 27 days post infection, respectively (p = 0.0037). Statistical significance was determined using GraphPad Prism version 4.0c for Mac, GraphPad Software, San Diego California, USA, www.graphpad.com; ** indicates p<0.01. (2.62 MB TIF) [file ppat.1000460.s001.tif]

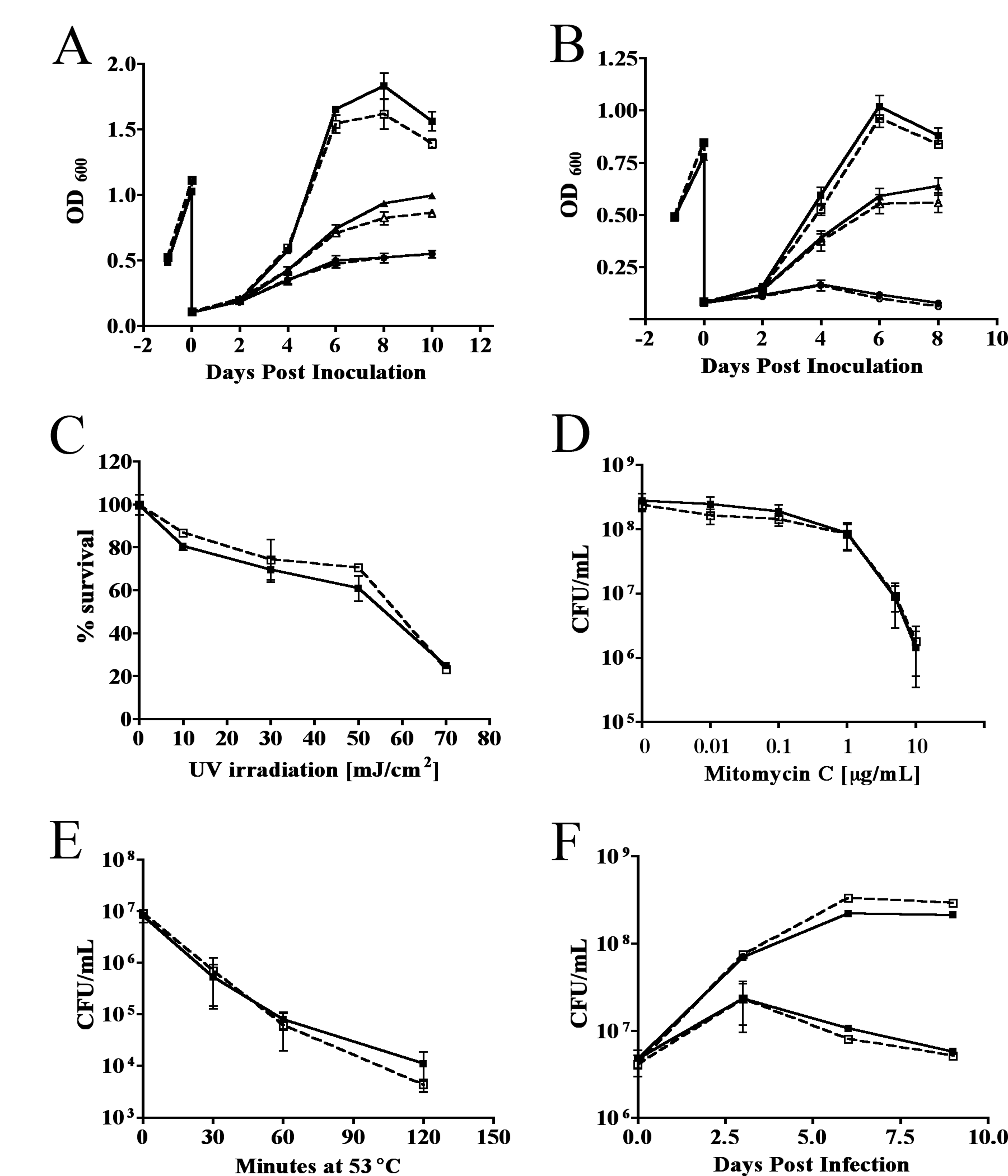

Supplement: Figure S2 — Characterization of the response of Δrv2623 to environmental stress. Optical density(OD600) was used to monitor the growth of Erdman (solid line) and Δrv2623 (dashed line) cultures in the presence of (A) superoxide anion (O2 −) following 1∶10 dilution of log-phase culture into fresh Sauton's media containing 10 µM (triangles) or 20 µM (circles) phenazine methosulfate (PMS) or (B) the Fe3+-dependent quinone antibiotic streptonigrin at concentrations of 0.01 µM (triangles) or 0.1 µM (circles). For (A,B): Untreated control cultures are represented by square symbols. (C) Sensitivity to ultraviolet irradiation was examined by exposing cells on solid media to the indicated energies and determining the number of surviving bacilli. Data are expressed as a percent survival relative to unexposed control plates for each strain. (D) The number of surviving CFU/ml was determined following treatment of log phase culture with increasing amounts of mitomycin C for 1 hour at 37°C. (E) Similarly, the number of surviving bacilli was determined following incubation at 53°C for the indicated times. (F) Log phase cultures were diluted 1∶10 into media at pH 7.0 or pH 4.0 and grown for several days and the number of surviving CFU/ml was monitored. Error bars represent the standard error of the means; each is a combination of at least two independent experiments performed in triplicate. (15.14 MB TIF) [file ppat.1000460.s002.tif]

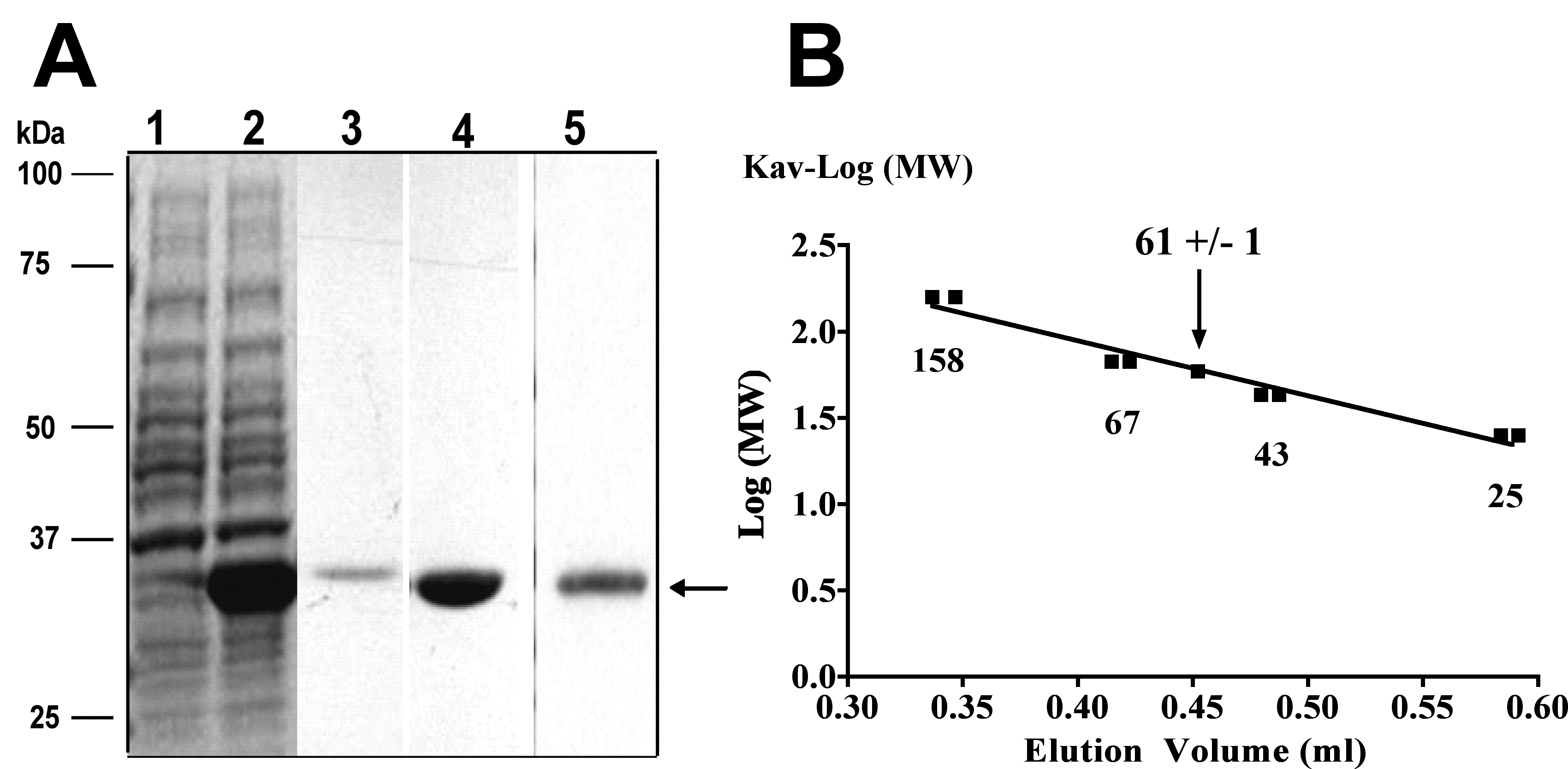

Supplement: Figure S3 — Purification and gel filtration profile of His6-Rv2623. (A) Expression and purification of His6-Rv2623. Lane 1: Uninduced pQE-rv2623E.coli cell lysate carrying expression construct pQE-rv2623; Lane 2: Induced pQE-rv2623E.coli cell lysate; Lane 3 and Lane 4: Purified His6-Rv2623: 0.3 mg and 3 mg, respectively; Lane 5: Western blot probed with anti-RGS-His antibody. Arrow indicates His6-Rv2623 (32 kDa). (B) Gel filtration profile of purified His6-Rv2623 eluted from a Superdex 200 10/300 GL column. Molecular weight (61 kDa) was determined based on the specific retention time corresponding to the His6-Rv2623 peak compared to molecular weight standards. The predicted molecular weight of Rv2623 is 32 kDa and purified protein is representative of a dimer of Rv2623. (5.69 MB TIF) [file ppat.1000460.s003.tif]

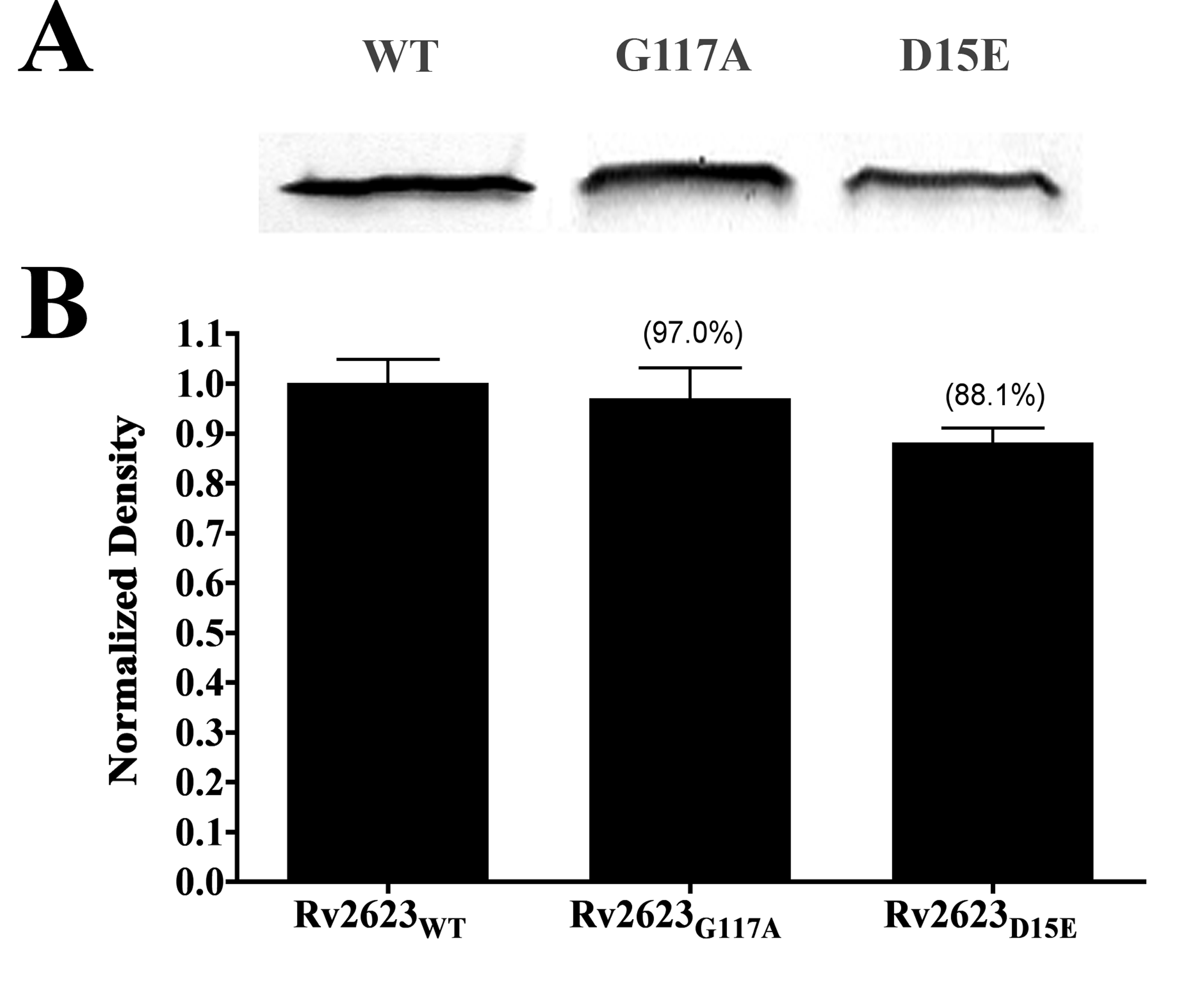

Supplement: Figure S4 — Relative expression level of wildtype(WT) and mutant Rv2623 in M. smegmatis. Strains of M. smegmatis that carried pMV261::rv2623 WT, pMV261::rv2623 G117A, and pMV261::rv2623 D15E overexpression plasmids were grown to late log phase in 7H9 media supplemented with kanamycin (40 µg/ml). The OD600 of the cultures was measured and used to estimate the number of CFU/ml (conversion factor: OD600 1 = 3×10^8^ CFU/ml). An equivalent number of cells for each strain were boiled for 5 minutes in denaturing sample loading buffer and separated by SDS-PAGE. (A) Western blotting techniques were used to detect Rv2623 in the total protein extract from these strains using monoclonal anti-Rv2623. (B) The immunoblot was scanned and analyzed with ImageQuant densitometry software (Molecular Dynamics). As different amounts of cells were boiled in different experiments, the relative intensity of the bands is expressed as a percent of the signal corresponding to Rv2623WT overexpression so that multiple experiments could be combined. The error bars represent the standard deviation and the percent of wild type Rv2623 expression is indicated above the bars. The data indicates that mutant Rv2623 overexpressed at similar levels compared to WT Rv2623. (4.75 MB TIF) [file ppat.1000460.s004.tif]
