# Supplementary material for: Mycobacterium tuberculosis Universal Stress Protein Rv2623 Regulates Bacillary Growth by ATP-Binding: Requirement for Establishing Chronic Persistent Infection
Source: PLoS Pathog. 2009 May 29;5(5):e1000460. doi: 10.1371/journal.ppat.1000460 (PMC2682197; doi:10.1371/journal.ppat.1000460)
Supplement: Protocol S1 — Semiquantitative scoring system for immunopathology of guinea pig tissues; and Crystallography: Summary of data collection and refinement statistics. (2.82 MB PDF) [file ppat.1000460.s005.pdf]

Protocol S1

Semiquantitative Scoring System for Immunopathology of Guinea Pig Tissues. Hematoxylin & Eosin-stained lung sections from guinea pigs infected with *M. tuberculosis* were scored individually based on the above system. The Total Lung Score for each animal represents the summation of the scores for each criterion; the maximum score for each animal is 24.

Percent of lung affected estimated at low magnification (2-4X obj.); scored as follows:

0 - no lesions in lung sections
1 - up to 25% of lung involved
2 - up to 50 % of lung involved
3 - Up to 75% of lung involved
4 - Above 75% of lung involved

Character of lesions were scores as follows:

Primary lesions

0 - No primary lesions present at section level
1 - A single primary lesion
2 - Two or more primary lesions, multifocal
3 - Two or more primary lesions, multifocal to coalescing
4 - Primary lesions, coalescing and extensive

Secondary lesions

1 - Up to 25% of lung involved
2 - Up to 50% of lung involved
3 - Up to 75% of lung involved
4 - Above 75% of lung involved

Necrosis

0 - No apparent necrosis present
1 - A single focus of necrosis present
2 - Two or more foci of necrosis, multifocal
3 - Two or more foci of necrosis, multifocal to coalescing
4 - Coalescing foci of necrosis that are extensive

Mineralization

0 - No mineralization present at section level
1 - A single focus of mineralization present
2 - Two or more foci of mineralization, multifocal
3 - Tow or more foci of mineralization, multifocal to coalescing
4 - Coalescing foci of mineralization that are extensive

Fibrosis (scored relative to the cellularity of primary and secondary lesions)

0 - No fibrosis present
1 - Minimal fibrosis present
2 - Equal area of cellularity and fibrosis
3 - More fibrosis than cellularity
4 - Extensive fibrosis with low cellularity

Maximum lung score: 24

Crystallography: Summary of Data Collection and Refinement Statistics.

**Space Group**	**$C222_1$**
**Unit Cell Parameters (Å)**	***a*** **= 173.0**
	b **= 241.5**
	c **= 241.7**
**Data Collection Statistics:**	
**Wavelength (Å)**	**1.08**
**Resolution (Å)**	**140- 2.9**
**Reflections measures**	**694890**
**Unique reflections**	**111196**
**$^{a}R_{merge}$ (%)**	**^{b}9.5(37.3)**
**Completeness (%)**	**99.2(98.4)**
**Refinement Statistics**	
**Total number of atoms**	**19011**
**Protein**	**18139**
**ATP**	**496**
**Mg^{2+}**	**16**
**Solvent**	**360**
**Average Thermal Factors ($Å^2$)**	
**Protein**	**47.6**
**ATP**	**32.8**
**Mg^{2+}**	**31.5**
**Solvent**	**41.7**
**RMS Deviation from Ideality**	
**Bond lengths (Å)**	**0.01**
**Bond Angles (°)**	**0.98**
**Ramachandran Plot Regions (%)**	
**Most favored**	**91.1**
**Additional allowed**	**8.3**
**Generously allowed**	**0.6**
**$^{c}R_{factor}$/$^{d}R_{free}$ (%)**	**25.0/26.8 (35.0/40.1)**

[a]R_{merge} (%) = $\Sigma_{hkl} \Sigma_i$ | I_{hkl} - $<I>_{hkl}$ | / $\Sigma_{hkl} \Sigma_i <I>_{hkl}$ x 100; where I_{hkl} is the intensity measurement of a given reflection and $<I>_{hkl}$ is the average intensity for multiple measurements of this reflection.

[b]Values in parentheses refer to the highest resolution bin.

[c]R_{factor} (%) = | Fo_{hkl} - Fc_{hkl} | / | Fo_{hkl} | x 100; where Fo_{hkl} is the observed structure factor amplitude and Fc_{hkl} is the calculated structure factor amplitude.

[d]R_{free} was calculated for 5 % of the diffraction data and monitored throughout refinement.
