# Supplementary material for: Mycobacterium tuberculosis Universal Stress Protein Rv2623 Regulates Bacillary Growth by ATP-Binding: Requirement for Establishing Chronic Persistent Infection
Source: PLoS Pathog. 2009 May 29;5(5):e1000460. doi: 10.1371/journal.ppat.1000460 (PMC2682197; doi:10.1371/journal.ppat.1000460)
Supplement: Table S1 — Gene list and accession numbers. (0.03 MB DOC) [file ppat.1000460.s006.doc]

**Table S1. Gene List and Accession Numbers**

| Genes mentioned in the paper | UniProtKB No. | Gene Name/organism |
| --- | --- | --- |
| *rv2623* | O06189 | *TB31.7* *Mycobacterium tuberculosis* |
| *dosR* | P95193 | *devR Mycobacterium tuberculosis* |
| *dosS* | P95194 | *devS Mycobacterium tuberculosis* |
| *E.coli uspA* | P0AED0 | *uspA* *Escherichia coli* strain K12 |
| *MJ0577* | Q57997 | *MJ0577 Methanocaldococcus jannaschii* |
| *dosT* | O53473 | *Rv2027c Mycobacterium tuberculosis* |
| *hspX* | P0A5B7 | *hspX Mycobacterium tuberculosis* |
| *H.influenzae uspA* | Q4QM85 | *uspA Haemophilus influenza strain 86-028NP* |
