# Supplementary material for: Mycobacterium tuberculosis Universal Stress Protein Rv2623 Regulates Bacillary Growth by ATP-Binding: Requirement for Establishing Chronic Persistent Infection
Source: PLoS Pathog. 2009 May 29;5(5):e1000460. doi: 10.1371/journal.ppat.1000460 (PMC2682197; doi:10.1371/journal.ppat.1000460)
Supplement: Text S1 — Supporting materials and methods. (0.03 MB DOC) [file ppat.1000460.s007.doc]

**Text S1**

**Supplemental Materials and Methods**

**Generation of *M. tuberculosis* ∆*rv2623*.** DNA regions that flank *rv2623* were amplified by the polymerase chain reaction (PCR) using primers ∆*rv2623*-UF-5’-G GTACCGAGACGACGACCACAT-3’ and ∆*rv2623*-UR-5’-TCTAGAGACATCGCGGTCCTCCT-3’ for the upstream flank [913 bp] and ∆*rv2623*-DF-5’-CTCGAGAGTCGCTGACTTAGGT-3’ and ∆*rv2623*-DR-5’-ACTAGTAGGAGAGCCGACGATG-3’ for the downstream flank [824 bp]. Flanking regions were then subcloned into multiple cloning sites on either side of a hygromycin cassette, which contains the *hyg* gene, conferring resistance to hygromycin, on the vector pYUB854. This allelic exchange construct was then used to generate recombinant mycobacteriophage for gene knockout from *M. tuberculosis* Erdman as described by Bardarov et al., 2002 (1).

For complementation studies, expression of *rv2623* was driven from two promoters including *hsp60* promoter, a constitutively expressed mycobacterial heat shock protein and endogenous *rv2623* promoter. The *rv2623* coding sequence was amplified by PCR using the following primers; forward: 5’-GAATTCATGTCATCGGGCAATTCATCTC-3’, reverse: 5’- AAGCCTCTAAGTCAGCGACTCGCGTGC-3’ and directionally subcloned into EcoRI/HindIII sites downstream and in frame with the *hsp60* promoter (P_hsp60_) on the integrative plasmid pMV361.kan. A promoterless variant of this vector, pMV306Kan, was utilized as a vehicle for expression of *rv2623* driven by its native promoter. Cloning of the *rv2623* gene including 347 bp of upstream regulatory sequences was done by PCR using the forward primer: 5’-GAATTCTTGTCACCGCTTCCATGGC-3’ and reverse: 5’- AAGCCTCTAAGTCAGCGACTCGCGTGC-3’ then inserted into EcoRI/HindIII sites of pMV306.Kan. For overexpression studies, the EcoRI-*rv2623*-HindIII PCR fragment was subcloned in frame with P_hsp60_ on the multi-copy plasmid pMV261.kan.

**Protein Expression and Purification.** Using the following primers: forward, 5’-CGGATCCATGTCATCGGGCAATTCATCTCT-3’, and reverse, 5’-CCTCGAGCTAAGTCAGCGACTCGCGTGCCA-3’, with BamHI and XhoI restriction sites respectively (underlined) for cloning purposes, the *rv2623* fragment was PCR-amplified, cloned into pCR-Blunt II-TOPO (Invitrogen life technologies) and confirmed by DNA sequencing (Genewiz). The *rv2623* gene fragment was first subject to cleavage by XhoI (New England Biolabs) and the 5’-overhang was then filled in by the Klenow Fragment. The fragment was next cleaved with BamHI (New England Biolabs) and finally subcloned into the corresponding BamHI and SmaI site of the expression vector pQE80L (Qiagen, Inc.), producing the plasmid pQE-*rv2623*.

Following induction of gene expression with 0.3 mM isopropyl beta-D-thiogalactoside (IPTG), *E. coli* BL21 (DE3) transformed with pQE-*rv2623* were incubated at 30 °C for 3 hours. Cells were then collected by centrifugation, resuspended in lysis buffer (50 mM NaH_2_PO_4_, 300 mM NaCl, 15 mM imidazole, pH 8.0) and lysed by sonication. The cleared cell lysate was then incubated with Ni-NTA agarose for 1 hour at 4 °C followed by several washes (50 mM NaH_2_PO_4_, 300 mM NaCl, 50 mM imidazole, pH 8.0). His_6_-Rv2623 was eluted from the beads (50 mM NaH_2_PO_4_, 300 mM NaCl, 250 mM imidazole, pH 8.0). The eluent was then dialyzed against 50 mM Hepes, 50mM NaCl, 1 mM MgCl_2_, 13 mM 2-mercaptoethanol, 10 % Glycerol (dialysis buffer) at 4 °C for overnight, then 3 hours.

**High Performance Liquid Chromatography (HPLC).** Nucleotide was separated from purified Rv2623 by boiling. For experiments that examined the nucleotide binding capacities of wildtype and mutant Rv2623, samples were then centrifuged at 14,000 rpm for 15 min and the supernatant was diluted into Buffer A (0.04 M NaH_2_PO_4_, pH 5.5) to a final volume of 0.5 ml. These samples were then loaded onto an analytical, anion-exchange HPLC column (AX300, Eprogen Inc.) and eluted isocratically at 1 ml/min using a 50:50 mixture of Buffer A and Buffer B (1M NaH_2_PO_4_, pH 5.5) (2). For stoichiometric studies, undiluted supernatants (or supernatants diluted within the linear range of a standard curve comprising known ATP concentrations in water) were analyzed using a Mono Q HR 5/5 column (Amersham Biosciences, presently GE Healthcare) according to published procedures (2) that were modified for rapid separation of adenine nucleotides AMP, ADP, and ATP.

**Crystallization.** Cube-like crystals with approximate dimensions of 0.2 x 0.2 x 0.2 mm appeared within one week at 4 ˚C under sitting-drop, vapour-diffusion conditions that consisted of a 1:1 v/v ratio of protein to mother liquor (29 % polyacrylate 5100, 100 mM sodium citrate, 20 mM MgCl_2_, 200 mM MES, pH 5.4). Crystals were prepared for cryogenic data collection by transfer to mother liquor diluted 1: 4 v/v with 100 % ethylene glycol.

**Site-Directed Mutagenesis of Rv2623.** The following PCR primers were used to incorporate mismatch point mutations into plasmid vectors bearing the *rv2623* coding sequence: **G117A:**GTGCTGATGGTCGTGG**C**TTGTCTCGGAAGTGGG and CCCACTTCCGAGACAA**G**CCACGACCATCAGCAC; **D15E**: TTATCGTCGGGATCGA**G**GATTCACCGGCCGCAC and GTGCGGCCGGTGAATC**C**TCGATCCCGACGATAA. Substituted nucleotide sequences are in bold type. The PCR reactions were digested with Dpn1 (New England Biolabs) for 1 hour and the reaction was then used to transform TOP10 *E. coli* (Invitrogen). Transformants were selected on LB agar supplemented with 40 µg/ml kanamycin (pMV261::*rv2623*) or 100 µg/ml amplicillin (pQE-*rv2623*). Plasmid DNA was prepared from these cells and recombinant *rv2623* sequences were confirmed by DNA sequencing (Genewiz). Confirmed recombinant *rv2623* expression vector DNA preparations were then used to transform the appropriate bacterial strains: *M. smegmatis* mc^2^155, *M. tuberculosis* and *E. coli* BL21 (DE3), yielding strains pMV261::*rv2623* and pQE-*rv2623*, respectively.

**Thermal Denaturation Analysis.** Thermal denaturation profiles for purified, wild type and mutant Rv2623 (diluted to 10 µM in 100 mM NaCl; 150 mM HEPES, pH 8.0) were determined using an IQ5 Real Time PCR Detection System (Bio-Rad) after a 2 hours, 4 ˚C preincubation with 5x Sypro Orange protein gel stain (Invitrogen). In this study, we measured an increase in fluorescence intensity at λ =575 nm (excitation λ= 485 nm) during a temperature ramp from 20 to 95 ˚C. These fluorescence changes arise from quantitative binding of Sypro Orange dye to hydrophobic surfaces that are exposed during temperature-dependent protein denaturation. The transition unfolding temperature (Tm), which is obtained from the first derivative maximum of the fluorescence intensity - temperature curve, provides a structural stability measure for our samples.

**Phylogenomic Analysis.** Phylogenomic analysis of the Rv2623 structure was guided by Bayesian Evolutionary Tree Estimation (BETE; available through the Berkeley Phylogenomics Group Phylofacts Resource, http://phylogenomics.berkeley.edu/phylofacts/) methodology.

**Reference**

1. Bardarov S, Bardarov S Jr, Pavelka MS Jr, Sambandamurthy V, Larsen M, et al. (2002) Specialized transduction: an efficient method for generating marked and unmarked targeted gene disruptions in *Mycobacterium tuberculosis*, *M. bovis* BCG and *M. smegmatis*. Microbiology 148:3007-3017.

2. Kremmer T, Paulik E, Boldizsar M, Holenyiu I (1989) Application of the fast protein liquid chromatographic system and MonoQ HR 5/5 anion exchanger to the separation of nucleotides. J Chromatogr 493:45-52.
